# A hitchhiker’s guide to cerebrospinal fluid biomarkers for neuro-oncology

**DOI:** 10.1093/neuonc/noae276

**Published:** 2024-12-30

**Authors:** Cecile Riviere-Cazaux, Michael B Keough, Jeffrey A Zuccato, Rahul Kumar, Sebastian C Schulz, Arthur E Warrington, Michael W Ruff, Benjamin M Ellingson, Nader Sanai, Jian L Campian, Sani H Kizilbash, Ian F Parney, Gelareh Zadeh, Mustafa Khasraw, Tobias Kessler, Ugur Sener, Daniel P Cahill, Alireza Mansouri, Terry C Burns

**Affiliations:** Department of Neurological Surgery, Mayo Clinic, Rochester, MN, USA; Department of Neurological Surgery, Mayo Clinic, Rochester, MN, USA; Division of Neurosurgery, Department of Surgery, University of Toronto, Toronto, Ontario, Canada; MacFeeters Hamilton Neuro-Oncology Program, Princess Margaret Cancer Centre, University Health Network and University of Toronto, Toronto, Ontario, Canada; Department of Neurological Surgery, Mayo Clinic, Rochester, MN, USA; Clinical Cooperation Unit Neurooncology, German Cancer Consortium (DKTK), German Cancer Research Center (DKFZ), Heidelberg, Germany; Department of Neurology and Neuro-oncology Program, Heidelberg University Hospital, Heidelberg, Germany; Department of Neurological Surgery, Mayo Clinic, Rochester, MN, USA; Department of Oncology, Division of Medical Oncology, Mayo Clinic, Rochester, MN, USA; Department of Neurology, Mayo Clinic, Rochester, MN, USA; Department of Radiological Sciences, David Geffen School of Medicine, University of California Los Angeles, Los Angeles, CA, USA; Ivy Brain Tumor Center, Barrow Neurological Institute, Phoenix, AZ, USA; Department of Oncology, Division of Medical Oncology, Mayo Clinic, Rochester, MN, USA; Department of Oncology, Division of Medical Oncology, Mayo Clinic, Rochester, MN, USA; Department of Neurological Surgery, Mayo Clinic, Rochester, MN, USA; Division of Neurosurgery, Department of Surgery, University of Toronto, Toronto, Ontario, Canada; Department of Neurological Surgery, Mayo Clinic, Rochester, MN, USA; Department of Neurosurgery, Duke University School of Medicine, Durham, NC, USA; Clinical Cooperation Unit Neurooncology, German Cancer Consortium (DKTK), German Cancer Research Center (DKFZ), Heidelberg, Germany; Department of Neurology and Neuro-oncology Program, Heidelberg University Hospital, Heidelberg, Germany; Department of Oncology, Division of Medical Oncology, Mayo Clinic, Rochester, MN, USA; Department of Neurology, Mayo Clinic, Rochester, MN, USA; Department of Neurological Surgery, Massachusetts General Hospital, Boston, MA, USA; Department of Neurosurgery, Penn State Milton S. Hershey Medical Center, Hershey, PA, USA; Department of Neurological Surgery, Mayo Clinic, Rochester, MN, USA

**Keywords:** biomarker, cerebrospinal fluid, glioma, monitoring, neuro-oncology

## Abstract

Cerebrospinal fluid (CSF) has emerged as a valuable liquid biopsy source for glioma biomarker discovery and validation. CSF produced within the ventricles circulates through the subarachnoid space, where the composition of glioma-derived analytes is influenced by the proximity and anatomical location of sampling relative to tumor, in addition to underlying tumor biology. The substantial gradients observed between lumbar and intracranial CSF compartments for tumor-derived analytes underscore the importance of sampling site selection. Moreover, radiographic features, such as tumor-CSF contact and blood-brain barrier disruption, are critical covariates that may affect biomarker detection and the abundance of plasma-derived analytes in CSF, respectively. Longitudinal intracranial CSF sampling, enabled by access devices like Ommaya reservoirs, may offer a window into treatment response and disease progression, though variability in analyte yield, sample volumes, and the dynamic effects of surgical resection pose challenges. This review critically evaluates the anatomic, radiographic, and longitudinal factors, or “time-space continuum,” that impact glioma CSF biomarker abundance. Practical considerations for longitudinal CSF biobanking, including access device placement and collection, are also reviewed. Key takeaways and recommendations for CSF glioma biomarker discovery and validation are provided as a “hitchhiker’s guide” based on our collective experience, along with resources for investigators aiming to develop CSF biobanking at their institutions.

Diffuse gliomas are primary brain tumors that inevitably recur, despite maximal safe surgical resection and aggressive chemoradiation.^1^ Longitudinal disease monitoring is typically performed using magnetic resonance imaging (MRI) in conjunction with the Response Assessment in Neuro-Oncology (RANO) criteria.^2^ However, prior treatment can make it difficult to distinguish between true disease progression and treatment-related changes via MRI.^3^ Despite urgent clinical need and numerous clinical trials, no therapy has significantly altered the standard-of-care for patients with high-grade gliomas (HGGs) in over 10 years.^4^ The Glioma Longitudinal AnalySiS (GLASS) Consortium has established that there is significant tumor evolution during treatment that impacts disease recurrence and therapeutic response.^5^ The reasons underlying therapeutic failures and disease evolution have been difficult to experimentally dissect, as one of the primary challenges in assessing disease burden and biological response in gliomas is re-accessing tumor tissue after the initial diagnostic sampling procedure.^6^ In contrast to other cancer types where serial tissue sampling is more routinely performed,^7–9^ longitudinal glioma sampling has historically not been frequently performed as it requires neurosurgical intervention via biopsy or resection that each carry a risk of adverse events.^10,11^ Although serial biopsies have been performed in a small subset of glioma clinical trials^12,13^ and are becoming increasingly endorsed within the drug development pipeline to assess pharmacodynamic impacts,^14^ cerebrospinal fluid (CSF) can provide complementary information to tissue in a more routinely accessible manner.

To address the need for better disease and pharmacodynamic monitoring, liquid biopsies have emerged as a promising alternative to serial tissue sampling.^15^ While plasma is easily acquirable, its sensitivity for detecting glioma-specific biomarkers has been generally limited due to the presence of the blood-brain barrier (BBB) and a high signal-to-noise ratio.^16–18^ CSF, in contrast, is a more proximal fluid to the tumor and can be accessed either pre-or-intra-operatively or longitudinally through a lumbar puncture (LP) or ventricular access devices such as Ommaya reservoirs or ventriculoperitoneal shunts. Several comprehensive reviews have detailed advances in glioma CSF biomarker discovery and validation across proteomics, cell-free DNA (cfDNA) genomics and methylomics, extracellular vesicle profiling, and other -omics approaches, with new findings continuing to emerge.^19–22^ In this review, we aim to focus on anatomic, radiographic, and longitudinal considerations, or the CSF “time-space continuum,” that affect disease monitoring and pharmacodynamic assessment—many of which are lessons derived from our own learning curve during the development of CSF biomarker studies. We provide case examples focusing on our collective experiences with proteomics, metabolomics, and cell-free DNA CSF studies, although significant work has also been performed in evaluating CSF extracellular vesicles, cytokines, and immune cells by other neuro-oncology teams.^23–27^ In addition to highlighting these key factors, we also share protocols and practical tips from our collective experience, offering guidance to researchers interested in developing CSF biomarker studies and biobanking at their institutions. By openly sharing these insights in this “hitchhiker’s guide,” our goal is to accelerate progress and enhance rigor in glioma CSF biomarker research as more teams undertake discovery and validation studies in both standard-of-care settings and clinical trials.

## Anatomic and Radiographic Considerations for Diagnostic and Monitoring CSF Biomarkers

### Overview: Flow of CSF and Methods of Acquisition

The bulk of CSF is thought to be primarily produced by the choroid plexus in the lateral ventricles, flowing through the foramen of Monro into the third and fourth ventricles, where additional CSF is generated.^28,29^ It then exits the foramina of Luschka and Magendie to enter the subarachnoid space, bathing and buffering the cerebrum and the spinal cord. CSF is then reabsorbed primarily through arachnoid villi at the superior sagittal sinus and other venous sinuses. Additional reabsorption occurs via lymphatic and glymphatic pathways,^30^ including when drainage via arachnoid villi is impaired.

Intracranial CSF can be sampled intra-operatively from ventricular or subarachnoid compartments. Ventricular CSF can be sampled longitudinally via a temporary external ventricular drain (proximal port or bag^31^) or permanent devices, including Ommaya or Rickham reservoirs^32^ or ventriculoperitoneal (VP) shunts.^33^ For longitudinal research CSF, Ommaya reservoirs can also be placed into resection cavities at the end of a clinically indicated resection (NCT04692337). Finally, lumbar CSF can be obtained via LP in the clinic or in the operating room (NCT04692324) at one or multiple timepoints.

### Impact of CSF Sampling Site on Biomarker Discovery and Validation

#### Lumbar Versus Intracranial CSF

**Figure 1.**
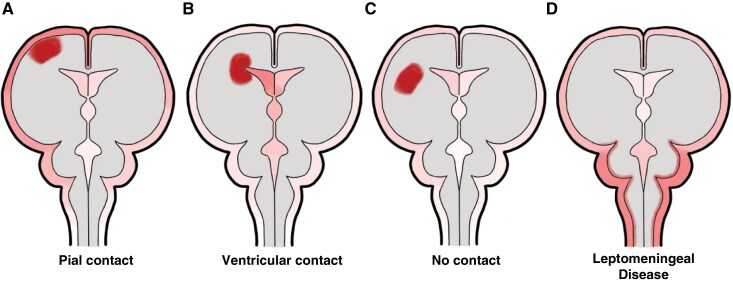
Biomarker distribution in CSF based on contact with CSF spaces. The relative concentration of biomarkers in CSF is depicted for tumors with (A) pial contact, (B) ventricular contact, (C) no contact (fully intraparenchymal tumors), and (D) leptomeningeal disease.

As most gliomas are supratentorial, there is a significant physical distance between where the tumor is centered and the lumbar spine CSF cistern, as accessed via LPs. After exiting the foramina of Luschka and Magendie, CSF flows both intracranially and into the spinal subarachnoid CSF space. Thus, except for tumors with ventricular contact, leptomeningeal dissemination, or those in the posterior fossa, CSF in the lumbar cistern is less likely to have been in significant contact with the tumor (Figure 1). It is possible that tumor-induced alterations of CSF flow,^34^ including ventricular trapping or hydrocephalus, could impact the relative CSF distribution of tumor-associated analytes along the neuraxis.^35^

**Figure 2.**
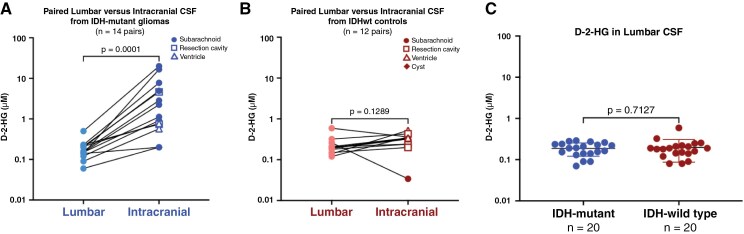
D-2-hydroxyglutarate (D-2-HG) as a candidate biomarker of IDH-mutant gliomas. (A) D-2-hydroxyglutarate (D-2-HG) concentrations were compared in paired lumbar and intracranial CSF samples from patients with IDH-mutant gliomas (*n* = 14 pairs), as well as in (B) IDH-wild-type tumors (glioma and other central nervous system tumors; *n* = 12 pairs), via a Wilcoxon matched-pairs signed-rank test. (C) D-2-HG concentrations were compared in lumbar CSF from patients with IDH-mutant gliomas versus IDH-wild-type tumors via Mann-Whitney test. The normality of the distribution was tested using a D’Agostino-Pearson normality test. Lines indicate the mean with standard deviation.

Multiple studies have demonstrated a gradient along the neuroaxis for metabolites and proteins, including glucose and immunoglobulins.^36–40^ For example, one study in normal pressure hydrocephalus (NPH) revealed a preferential distribution of specific proteins for intracranial CSF over the lumbar cistern, although a significant number of proteins remained similar between compartments.^37^ These analyses often leverage clinical treatment scenarios for conditions like NPH or meningitis, where diagnostic LPs are followed by therapeutic ventricular CSF diversion, enabling paired comparisons of lumbar and ventricular CSF within the same subjects. To obtain paired lumbar and intracranial CSF in patients with gliomas, LPs can be performed intra-operatively prior to patient positioning. Similar to non-glioma studies, such paired samples demonstrate a significant number of proteins that are more abundant in intracranial than lumbar glioma CSF, likely representing the impact of increased proximity to tumor. For tumor-specific candidate biomarkers like D-2-hydroxyglutarate (D-2-HG) in IDH-mutant gliomas, Kalinina et al.^41^ demonstrated a higher concentration of D-2-HG in cisternal than ventricular CSF for IDH-mutant gliomas, both of which were higher than that of lumbar CSF. Similarly, IDH1 mutant mRNA from extracellular vesicles was more abundantly detected in cisternal than lumbar CSF samples.^25^ Notably, CSF samples in these analyses were unpaired, so the possibility that some compartmental differences are due to other covariates across patients, such as extent of tumor contact with CSF, cannot be excluded. Nevertheless, our own findings in paired intracranial versus lumbar CSF from patients with IDH-mutant gliomas corroborate these findings, demonstrating significantly higher intracranial D-2-HG (*P* = .0001, Figure 2A), unlike in IDH-wild-type controls (*P* = .1289, Figure 2B) (C. Riviere-Cazaux, T. Burns, personal communication, November 5, 2024).

Despite these differences between the abundance of candidate biomarkers in lumbar versus intracranial CSF, Miller et al.^42^ found that cfDNA genomic profiles maintained concordance across sampling locations in six paired lumbar and ventricular CSF samples. Indeed, when sufficient cfDNA is detected in lumbar CSF for sequencing, known tumor-derived mutations can be detected,^42–49^ providing potential utility for glioma diagnosis and monitoring. Feasibility survey studies have demonstrated a very high likelihood of concordant diagnostic DNA mutational alterations in sampled CSF versus the gold standard of primary tumor tissue, approaching 80% sensitivity (comparing cfDNA detectable alterations versus primary tissue detectable alterations).^50,51^ Kalinina et al. concluded from three lumbar IDH-mutant glioma CSF samples that IDH-mutant gliomas could be distinguished from wild-type cases based on D-2-HG abundance.^41^ In contrast, our own data to date in a larger patient cohort suggest that D-2-HG is not more abundant in lumbar CSF from IDH-mutant than wild-type patients (*n* = 20 patients/group; *P* = .7127), suggesting further work is needed to determine the impact of clinical and anatomical covariates (Figure 2C) (C. Riviere-Cazaux, T. Burns, personal communication, November 5, 2024). Thus, lumbar CSF may still be useful for biomarkers like cfDNA that could be deployed for minimally invasive diagnosis, where the primary requirement is detecting enough of the analyte for downstream analyses, particularly for tumors with CSF contact. However, because lumbar CSF generally contains lower concentrations of tumor-derived analytes compared to intracranial CSF, it may be less effective for biomarkers where precise concentration levels are critical for distinguishing glioma from controls, or for evaluating changing concentrations over time during disease monitoring, particularly with the limited practicality of performing many serial LPs.

**Figure 3.**
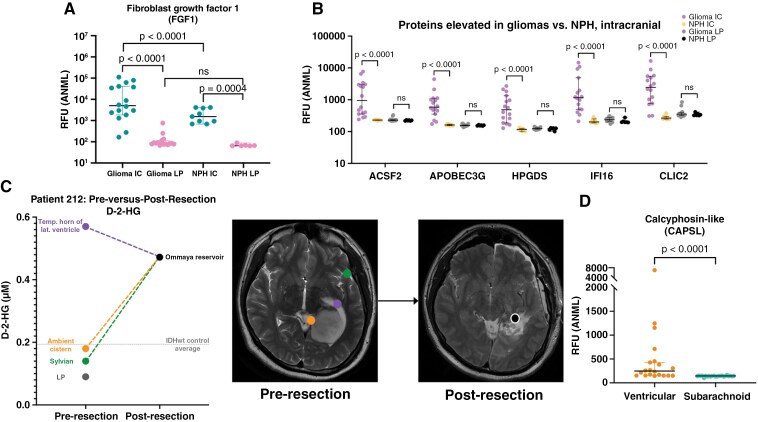
Location of CSF acquisition impacts glioma biomarker discovery. (A) FGF1 abundance is shown in intracranial and lumbar CSF from patients with gliomas (paired; *n* = 16), as well as from patients with NPH (unpaired lumbar, *n* = 6, and intracranial, *n* = 9). Wilcoxon signed-rank test was performed on paired glioma intracranial versus lumbar samples; all other tests performed were Mann-Whitney U tests. (B) The abundances of five of the most differentially abundant proteins between intracranial glioma versus NPH CSF are shown; lumbar glioma versus NPH CSF results are also shown (Mann-Whitney U tests). The glioma and NPH LP and intracranial sample sizes are as in (A). (C) CSF was obtained intra-operatively from a patient with a grade 2 IDH-mutant astrocytoma from the Sylvian fissure, temporal horn of the lateral ventricle, and ambient cistern, as well as the lumbar cistern via an LP. Post-resection CSF was obtained on POD 2 via an Ommaya reservoir implanted at resection. D-2-HG was quantified in each sample. (D) A Mann-Whitney U test was performed on the abundance of calcyphosin-like protein in ventricular (*n* = 20) versus subarachnoid (*n* = 23) CSF from patients with GBM. Lines indicate the median and 95% confidence intervals for each group. ACSF2 = acyl-CoA synthetase family member 2, APOBEC3G = apolipoprotein B mRNA editing enzyme catalytic subunit 3G, HPGDS = hematopoietic prostaglandin D synthase; IFI16 = gamma-interferon-inducible protein 16; CLIC2 = chloride intracellular channel protein 2.

Finally, as some analytes may be more abundant in one CSF compartment than another, it is crucial to compare anatomically matched CSF samples to avoid mistakenly identifying location-associated analytes as glioma-specific. For example, comparing intracranial glioma CSF to lumbar NPH samples might suggest that fibroblast growth factor-1 (FGF1) is elevated in gliomas (Figure 3A) (C. Riviere-Cazaux, T. Burns, personal communication, November 5, 2024). However, paired intracranial and lumbar glioma CSF analyses demonstrate that FGF1 is more abundant in intracranial than lumbar CSF in gliomas, as it is in intracranial versus lumbar CSF in NPH. This indicates FGF1 levels in part reflect the fact that this protein is an intracranial CSF-associated protein as opposed to a purely glioma-specific readout. Additionally, glioma-associated analytes identified in anatomically matched control samples may not be elevated in lumbar CSF, even within the same patient. For example, comparing intracranial glioma CSF to intracranial NPH CSF revealed several proteins elevated in glioma CSF that were not significantly elevated when lumbar glioma CSF was compared to lumbar NPH (Figure 3B) (C. Riviere-Cazaux, T. Burns, personal communication, November 5, 2024). Therefore, we propose that obtaining anatomically matched CSF samples is essential for systematic accuracy in glioma biomarker discovery and validation.

#### Ventricular Versus Subarachnoid Intracranial CSF

Intracranial CSF can be acquired from a ventricle, cistern, or from a sulcus (if a small incision is made in the arachnoid mater). CSF is produced in each ventricle and is exposed to different tissue as it flows from the ventricles to the subarachnoid space. As such, all intracranial CSF compartments are not equal in fluid composition. Indeed, Kalinina et al.^41^ demonstrated significant differences in cisternal versus ventricular D-2-HG across unpaired IDH-mutant samples, which we also identified across different intracranial compartments within a patient (example patient, Figure 3C) (C. Riviere-Cazaux, T. Burns, personal communication, November 5, 2024). Similarly, CSF proteomic studies revealed differences in the distribution of proteins across subarachnoid versus ventricular GBM CSF,^52^ as exemplified by a increased abundance of calcyphosin-like protein in ventricular CSF where it is produced by ependymal cells (Figure 3D). Moreover, tumor-associated cystic fluid is often more concentrated than CSF, such as with D-2-HG and cfDNA, which were 118.6x and 104.2x more abundant in pre-operatively sampled cyst fluid than a sulcus adjacent to tumor, respectively.^53,54^ Finally, CSF proteomics has also demonstrated that resection cavity fluid displays significant compositional differences to ventricular or subarachnoid CSF, presumably due in part to evolving, long-lasting post-operative gliosis.^52^ Fluid recovered from closed resection cavities differs from that obtained from those in contact with the lateral ventricles, with generally higher abundance of tissue-derived proteins. As such, the origin of intracranial CSF warrants consideration when performing biomarker discovery and validation, particularly in relation to the location of the tumor. Additionally, the volumetric yield from subarachnoid CSF can vary from 100 μL to over 5 mL, which can impact the type and number of analyses performed. Anatomical compartment limitations notwithstanding, we recommend sampling at least 100 μL at baseline for proteomics/metabolomics analyses and >2 mL for cfDNA-based analyses. However, we biobank all available volumes, even as low as 30-50 μL and up to 20 mL for research LPs and CSF access device taps, per our approved research protocols. Our protocols do not specify a maximum limit for intra-operative intracranial CSF samples obtained during resection, as long as there is no clinical contra-indication.

#### Key Takeaways and Recommendations: Lumbar Versus Intracranial CSF

Key takeaways and recommendations related to CSF sampling sites are presented in Table 1. Importantly, it should be noted that despite intracranial CSF being richer in glioma biomarkers, a relatively small number of well-powered studies have been performed with lumbar glioma CSF samples. As such, it is still possible that certain tumor-derived proteins or metabolites could exhibit differential abundances in glioma versus control lumbar CSF samples, detectable by more sensitive techniques. Moreover, the number of paired lumbar versus intracranial samples across studies is admittedly small, although this is offset by a large effect size, and requires further evaluation across different candidate biomarker subtypes, such as profiles of extracellular vesicles and immune cells. Additionally, for concentration-based biomarkers, such as 2-HG, location-specific concentration thresholds may need to be defined.

**Table 1.** Overview of Takeaways and Integration of CSF Biomarkers in Neuro-oncology.

Key takeaways for glioma CSF biomarker discovery and validation
CSF sampling sites
Intracranial CSF is richer in glioma biomarkers due to tumor proximity, but lumbar CSF remains clinically relevant for diagnostic biomarkers like cfDNA, where the focus is on obtaining sufficient analytes for analysis rather than relying on concentration differences to distinguish glioma from controls. Paired lumbar and intracranial CSF samples can be obtained intra-operatively to better define how and with which biomarker type to utilize each CSF source. When possible, CSF should be acquired from multiple intracranial CSF sampling sites to define differences in subarachnoid versus ventricular CSF. Differences between anatomical compartments for various -omics need to be better characterized. Comparing glioma CSF to anatomically matched controls is essential to avoid location-related bias in biomarker discovery.
Tumor-CSF contact and the blood-brain barrier
Tumor-CSF contact helps predict the likelihood of detecting glioma biomarkers, though better quantitative and modeling methods are needed. BBB disruption increases plasma-derived proteins in CSF, some of which may overlap with tumor proteins. Further work is needed to directly correlate the extent of BBB disruption with its impact on different CSF -omics. Blood contamination, in addition to BBB disruption, can introduce plasma proteins into CSF. Measuring hemoglobin, red blood cell count, or albumin quotient can help identify the effects of BBB disruption and contamination on CSF composition, although further work needs to be done to demonstrate the ability to deconvolute these two factors using such measures.
Longitudinal CSF for monitoring and pharmacodynamic biomarkers
Collaboration across institutions is essential for developing standardized CSF acquisition protocols defined by a consortium, data sharing (both -omics and clinical metadata), and conducting future prospective biomarker validation studies. Longitudinal intracranial CSF sampling is feasible with access devices like Ommaya reservoirs and aids in pharmacodynamic and monitoring biomarker discovery when acquired alongside MRIs. Resection has a significant, lasting impact on the composition of CSF independent of changes in tumor burden, impacting the identification of monitoring biomarkers. Further work is needed to evaluate the impact of resection across -omics.
How and when to incorporate glioma CSF biomarkers?
With plasma
Plasma is more accessible for serial collection, but CSF shows higher sensitivity for glioma detection, especially when there is tumor-CSF contact. Paired plasma and CSF samples are encouraged for longitudinal studies to determine which source is more reliable for specific biomarkers and to cross-validate results, enhancing confidence in disease monitoring. Further work is needed to more extensively directly compare these two liquid biopsy sources.
With tissue
Tissue remains the gold standard for diagnosis of primary or recurrent disease, but serial biopsies for monitoring are rarely performed. Longitudinal CSF paired with tissue results are crucial for validating biomarkers, particularly in identifying monitoring and pharmacodynamic biomarkers. Few studies to date have correlated longitudinal CSF paired to tissue, with substantial further work needed in this area.
With other CSF biomarkers
Integration of multi-omic biomarker data may increase confidence in disease monitoring results. However, to avoid integrating non-tumor variables like plasma proteins, selecting features present in tissue may enhance the reliability and generalizability of CSF biomarker models. Further work needs to be done to evaluate this idea across different -omics in CSF and tissue during standard-of-care or clinical trials.
With survival, imaging, and clinical practice
Despite limitations, imaging and survival remain the standard clinical measures for assessing glioma disease trajectory. When both measures agree with one another, candidate CSF biomarkers can be benchmarked against these metrics before clinical deployment. For future clinical integration, CSF biomarkers should be integrated at key points in the disease trajectory, such as distinguishing progression from treatment effects and assessing early pharmacodynamic impact at clinical trial initiation. Advanced imaging methods like ^18^F-DOPA PET, DWI, and DSC show promise for greater glioma detection sensitivity but are not yet part of RANO. Few studies to date have integrated CSF biomarkers with these imaging techniques and radiomics to determine if this could enhance disease monitoring accuracy.

*As applicable per section, if multiple statements are made, the takeaways and summaries below are ranked according to the level of evidence presented in the review, with the highest rank having the most evidence to date and the lowest requiring the greatest extent of further evaluation. Areas of suggested further work are Included, although these recommendations are not exhaustive and additional future directions are included in the review.*

### More than the Zip Code: Tumor-CSF Contact and Blood-Brain Barrier Disruption

#### Impact of Tumor-CSF Contact on Biomarker Abundance

Analyte diffusion occurs at ventricular ependymal surfaces or cortical pial surfaces in contact with ventricular or subarachnoid CSF, respectively (Figure 1). Multiple groups have correlated radiographic tumor-CSF contact with improved detection of tumor-associated CSF biomarkers.^42,43,46^ Wang et al.^46^ found that all thirteen contrast-enhancing HGGs abutting a CSF space had detectable lumbar CSF cfDNA. Of the five patients in their series who had lesions completely encapsulated by tissue (none of whom had HGG), none had measurable cfDNA. Subsequent data in a larger series of 85 glioma patients also found that CSF contact were significantly associated with improved cfDNA detection.^42^ As CSF contact can be variably defined and reported as a binary, rather than continuous, the quantitative correlation between CSF contact and biomarker detection remains to be determined.

Similar correlations between biomarker abundance and CSF contact have been observed in patients with brain metastases.^51,55^ However, intracranial CSF in these metastatic cases is typically collected only when clinically indicated, such as during shunt placement due to hydrocephalus from leptomeningeal disease (LMD)^56^ or at resection, where metastases have a predilection for the gray-white matter junction that is closer to pia.^57^ As a result, intracranial CSF samples from patients with brain metastases are frequently biased toward those with increased tumor-CSF contact,^58^ increasing the likelihood of identifying tumor-derived analytes in CSF (Figure 1D). This contrasts with gliomas, which, despite being diffusely infiltrative, are intrinsically intraparenchymal and less likely to have leptomeningeal dissemination. Differences in sampling contexts between brain metastases and gliomas must be carefully considered when comparing biomarkers across these tumor types, as certain differences in analyte abundance may be due largely to differences in CSF contact or proximity.

#### Radiographic Contrast Enhancement: Impact on Biomarker Abundance

BBB disruption occurs in most HGGs and is visualized by contrast enhancement on MRIs.^59^ The BBB limits tumor-derived analyte diffusion into plasma, with multiple studies revealing that focused ultrasound-induced BBB disruption increases candidate biomarker abundance in plasma.^16–18^ The relative burden of contrast-enhancing tumor also correlates with the detection of tumor-associated mutations in plasma cfDNA.^60,61^ In contrast, emerging studies demonstrate that contrast-enhancing tumor volume does not correlate with improved detection of glioma-associated cfDNA in CSF.^42,46^ For instance, in Wang et al.,^46^ contrast enhancement did not correlate with cfDNA detection in lumbar CSF from 35 primary CNS malignancies, including 14 HGGs, consistent with findings from Miller et al. in 85 glioma patients.^42^ Thus, since CSF is beyond the BBB, its disruption may be less relevant for detecting glioma cfDNA in CSF than plasma.

Interestingly, despite the lack of correlation between contrast-enhancing tumor volume and cfDNA detection, all four low-grade (presumably non-enhancing) gliomas with CSF contact in Wang et al.^46^ lacked detectable cfDNA. This contrasts with Miller et al.,^42^ where tumor grade did not significantly impact cfDNA abundance in the 85 gliomas studied, of which 39 were LGGs. Further work in larger cohorts is needed to understand how CSF contact, contrast enhancement, and tumor grade interact with each other and other factors to impact CSF biomarker detection, include that of hallmark biomarkers like IDH or H3K27M. Additional variables of interest include, but are not limited to, tumor cellularity, treatment status, and the balance between proliferation and apoptosis, given that cell death is necessary for cfDNA release.^62^ These variables likely differ across tumor-derived analytes and will require further investigation for each specific candidate biomarker.

#### Radiographic Contrast Enhancement: Impact on CSF Composition

BBB disruption impacts CSF composition by allowing analyte diffusion from plasma into the CNS, particularly for proteins and metabolites. Plasma-derived proteins are significantly enriched in glioma CSF at the time of maximal tumor burden.^52^ Mikolajewicz et al.^58^ independently demonstrated that their intracranial GBM CSF proteomic signature was associated with BBB disruption and angiogenesis. BBB disruption, as measured by CSF to serum albumin concentration ratios (Q_alb_) also significantly contributed to the lumbar CSF proteome in contrast-enhancing GBM, lymphoma, and brain metastases with LMD in Schmid et al.,^63^ with increased Q_alb_ correlating with worsened prognosis in GBM. Prior glioma-associated proteins have included vascular endothelial growth factor, beta-2-microglobulin, albumin, carbonic anhydrases (i.e. CA2, CA12), and other proteins, that are also plasma-associated.^64–66^ Similarly, CSF metabolomic studies report increased levels of carnitines and aminobutanal that are also likely to originate from plasma.^67,68^ Consistent with this notion, we had previously noted that plasma-derived metabolites are significantly more enriched in interstitial fluid from contrast-enhancing regions of HGGs when compared to non-enhancing regions.^69^ Specific to cfDNA, plasma-derived germline DNA may contaminate results, which can be evaluated via paired CSF and plasma samples. Plasma-derived proteases, nucleases, and other enzymes may impact the stability of some analytes depending on the analysis method utilized. Further investigation is required to deconvolute the contributions of BBB disruption versus tumor production to CSF composition—recognizing that some analytes may be shared between plasma and glioma cells. Independence of BBB disruption may need to be confirmed for any glioma-derived biomarker at the time of validation.

#### Key Takeaways and Recommendations: Tumor-CSF Contact and BBB Considerations

Tumor-associated CSF analyte abundance and composition are significantly impacted by the extent of tumor-CSF contact and BBB disruption, respectively. Additional takeaways and recommendations are provided in Table 1.

## Longitudinal Considerations: Implications for Disease Monitoring

### Longitudinal CSF: Current Status

**Figure 4.**
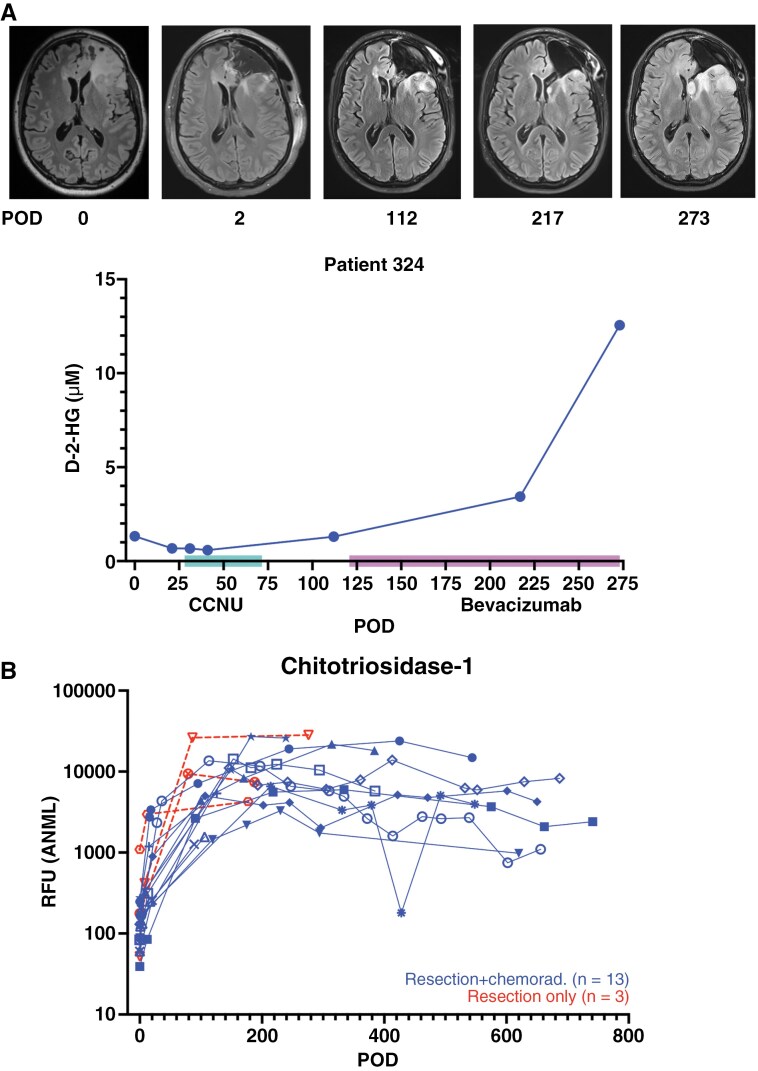
Longitudinal intracranial glioma CSF: impacts of progression and resection. (A) CSF was obtained intra-operatively from the lateral ventricle during resection for a recurrent grade 4 IDH-mutant astrocytoma, during which an Ommaya reservoir was placed for longitudinal CSF access. CSF was then collected at multiple points during treatment with lomustine (CCNU) and bevacizumab, with disease progression occurring near POD73 and 217. (B) The abundance of chitrotriosidase-1 was evaluated in longitudinal CSF samples from patients with gliomas beginning at the time of resection (POD0), 13 of whom underwent chemoradiation and 3 of whom only underwent resection.

Clinical MRIs are standard-of-care for disease monitoring, but can frequently be confounded by treatment-induced radiographic changes mimicking disease progression,^3^ especially with immunotherapy^70^ and after chemoradiation.^71^ Steroids or bevacizumab also render response assessment challenging, as they temporarily reduce radiographic contrast enhancement.^72^ Advanced imaging has not yet been integrated into clinical practice due to its limited availability and the single-center, non-randomized nature of the studies. Recently, we and others have utilized CSF access devices to obtain longitudinal intracranial CSF samples during standard-of-care and experimental therapies.^52,53,54,73,74^ Compared to repeated LPs, systematic intracranial CSF collection is better tolerated. Studies have identified proteomic and metabolomic signatures of resection,^52^ decreasing tumor-associated variant allele frequency (VAF) with cytoreduction,^54^ and, specific to IDH-mutant gliomas, increasing D-2-HG with disease progression (Figure 4A) (C. Riviere-Cazaux, T. Burns, personal communication, November 5, 2024). Additionally, CSF has been used for pharmacodynamic evaluation of pembrolizumab,^52^ bevacizumab,^52^ CAR-T cells targeting EGFR and IL-13Rα2,^73,74^ and GD-2 directed CAR-T cells^75,76^ in gliomas, including analysis of cytokines such as interleukins-2 or 6 and interferon-Ɣ in response to immunotherapies.

### Impact of Intracranial CSF Locations on Longitudinal CSF

Intra-operative CSF may be acquired from a different location than post-operative CSF from an access device. As not all resections will reach the ventricles, subarachnoid CSF may be sampled more frequently than ventricular CSF. For reservoirs placed at resection, longitudinal CSF will originate from the resection cavity, with or without ventricular contact. CSF circulation from ventricular contact also impacts CSF composition in the resection cavity. Ventricular CSF is sampled if the Ommaya is placed ventricularly (i.e. at the time of biopsy prior to resection, NCT06322602) or if obtained from a shunt. Of note, contact of the ventricle with the resection cavity likely alters the composition of the ventricular fluid as compared to pure ventricular fluid, given the impact of post-surgical changes.

Differences across intra-operatively sampled CSF compartments may impact the relative change with resection (example patient with D-2-HG, Figure 3C) (C. Riviere-Cazaux, T. Burns, personal communication, November 5, 2024). After surgery, CSF sampling location remains consistent, although evolving changes in the resection cavity are reviewed later. Thus, locational differences in pre-versus-post-resection CSF should be considered when determining if a candidate biomarker decreases with resection. When possible, intra-operative sampling across multiple CSF compartments may reveal whether a candidate biomarker decreases depending on the baseline sample utilized.

### Impact of Resection on CSF-Omics: Learning Through Experience

While CSF is a promising monitoring biomarker source, resection has a profound impact on CSF composition, independent of changes in glioma burden.^52,54^ Longitudinal CSF proteomics revealed dynamic, evolving changes that were present months after surgery,^52^ which confounded our ability to identify a chemoradiation-associated proteomic signature. Indeed, we initially identified a highly conserved pre-versus-post-chemoradiation signature across patients in both discovery and validation analyses, characterized by elevated chitotriosidase-1 (*P* = .0078), which we hypothesized was due to radiation-induced senescence. However, when we evaluated similar time points for three patients who did not undergo chemoradiation, their post-resection CSF proteome was nearly identical to that of the chemoradiation patients, exemplified by CHIT1 (Figure 4B). The signature that we had initially misattributed to radiation was instead one of evolving post-operative changes. Further analysis demonstrated that sets of proteins follow different trajectories after resection, including the increased abundance of inflammatory cytokines early after surgery. Thus, the impact of resection independent of glioma should be evaluated when studying candidate monitoring or pharmacodynamic biomarkers. Multiple single-arm studies delivering CAR-T cell therapies have reported longitudinal inflammatory impacts in CSF.^73–75,77^ Parallel post-surgical cohorts without immunotherapy are needed to confirm that pharmacodynamic results are not confounded by surgery. Immune cell profiling in CSF, previously performed in brain metastases,^26^ may also be of interest as orthogonal evidence to cytokine/chemokine findings with resection versus immunotherapies.

### Implications of Variable Volumes for Longitudinal CSF Monitoring

Volume requirements for CSF -omics will vary across platforms, with small volumes (100 μL) typically sufficient for metabolomics and proteomic studies, and larger volumes (>2 mL) for cfDNA-based profiling. We aim to collect a consistent volume (up to 20 mL) across taps to minimize variability in longitudinal CSF acquisition and allow for extensive multi-omic profiling. However, resection cavities may collapse over time, particularly if there is no ventricular contact. In extreme cases, the catheter could become lodged in parenchyma, essentially yielding very small volumes of interstitial fluid. Gliosis and scarring may progressively isolate the resection cavity, particularly if there is no ventricular communication. If collected volumes decrease post-operatively and if there is less CSF flow, tumor-derived analyte concentration may increase longitudinally, erroneously suggesting tumor recurrence. Accordingly, longitudinal CSF volumes should be documented for correlation with MRIs and analyte concentrations. Moreover, studies in lumbar NPH CSF demonstrate elevated immunoglobulin and albumin concentrations in the initial CSF fraction compared to later fractions collected from upstream CSF.^39^ A similar principle could apply to intracranial CSF sampling, although this hypothesis remains to be tested. When anatomy permits, we recommend collecting equal volumes at each time point that can then be divided into smaller aliquots to enable multi-omic analysis without introducing freeze-thaw cycles,^19,78^ as these cycles can adversely impact the detection and discovery of glioma-derived biomarkers depending on the analyte’s relative stability (example case, Supplementary Figure 1) (C. Riviere-Cazaux, T. Burns, personal communication, November 5, 2024).

#### Case Example: Pitfalls of Longitudinal Interpretation—CSF cfDNA

Optimal monitoring via CSF requires minimal variability in factors other than glioma burden, with stable values obtained over time in the setting of stable disease. Standardized pre-analytical workflows minimizing the possibility for contamination are also essential. However, candidate biomarkers can be impacted by several factors not directly associated with changing tumor burden. To illustrate this experience, we will again utilize tumor-derived cfDNA due to its unambiguous origin in glioma.^54^ Longitudinal cfDNA yield can fluctuate substantially, including to levels inadequate for next-generation sequencing (NGS), resulting in missing data during disease monitoring. Moreover, cfDNA originates from both tumor and stroma.^79^ High stromal-derived cfDNA abundance may occur early after resection or with treatment-related inflammation exacerbated by radiation necrosis or immunotherapies. Increased stromal cfDNA relative to tumor cfDNA can artificially decrease changes in tumor-associated VAF or copy number burden. Further work will be needed to understand variables impacting longitudinal analyte yield and how to appropriately account for variable non-tumor cfDNA contributions.

Moreover, tumor cells must die for cfDNA to be released, which occurs both with cell turnover in proliferative disease and during the initial response to an effective therapy, followed by successful cytoreduction. Indeed, in plasma cfDNA, mutational VAFs transiently increase after cytoreduction before normalizing during stable disease.^80–82^ During treatment, changes in cfDNA levels between time points may provide a better indication of relative disease activity rather than overall burden. Once cfDNA levels stabilize, deviations during surveillance could detect increased disease activity indicative of progression. This principle may also apply to other non-cfDNA glioma biomarkers, though further work is needed to identify such monitoring biomarkers.

### Technical Considerations for Research Ommaya Placement and Tapping

**Figure 5.**
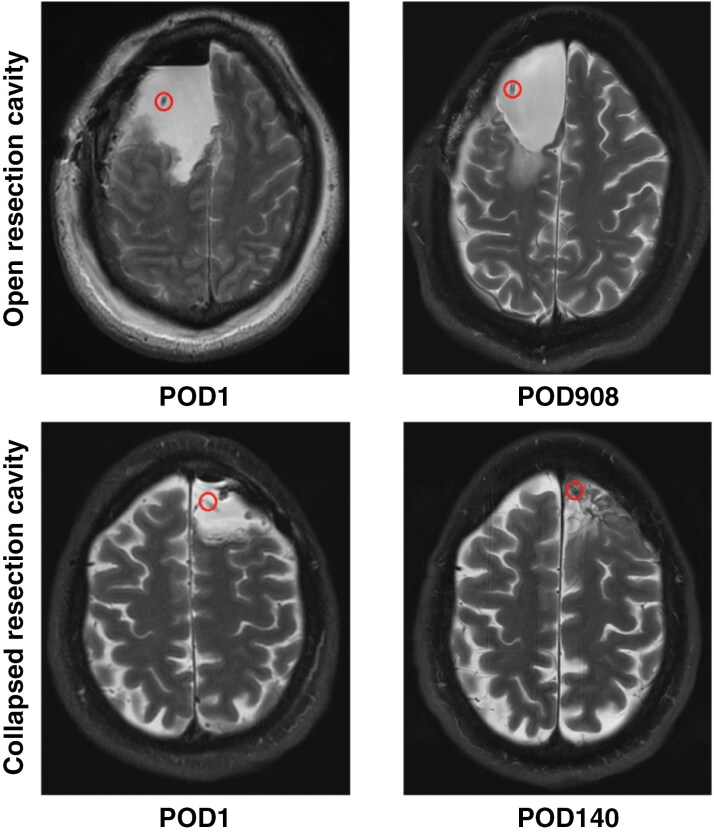
Ommaya reservoir catheters in the resection cavity. The location of the ventricular catheters attached to Ommaya reservoirs over time is shown in open (left) versus collapsed (right) resection cavities from two patients on T2-weighted MRI scans. The catheter tip is circled.

Reservoirs implanted in the resection cavity may or may not be continuous with the ventricles depending on an extent of resection. Catheter length and orientation should be carefully considered to keep the tip away from parenchyma that could obstruct CSF access (Figure 5). For tumors without ventricular contact, the benefits versus limitations of catheter placement within the ventricle (further from the tumor) versus cavity should be weighed. If cavity collapse is likely, ventricular placement may be advised to ensure longitudinal CSF acquisition with stable composition and yield. While both methods are reasonable, they result in different and non-comparable compartmental access. Implantation of both an intraventricular catheter and intra-cavitary catheter in the same patient could be considered to directly compare the two methods, although the risks of infections must be considered. Post-operative imaging can document the catheter tip location, which may predict the volume yield. If the cavity is largely collapsed, the reservoir should not be depressed to allow recovery of fluid pooled within it. Documentation of prior low volume is recommended to make providers aware of which patients have collapsed cavities.

Moreover, the risks of the research Ommaya reservoir placement for longitudinal CSF acquisition must be acknowledged. The primary risk for reservoir placement is infection, which ranges from 8% to 15% in current literature.^83–85^ The risk of hemorrhage with reservoirs or shunts is approximately 1% if tissue is penetrated (i.e. at biopsy),^85^ which is mitigated when placed in the resection cavity. Sample protocols are provided in Supplementary materials that can be adapted at the physician’s discretion.

### Key Takeaways and Recommendations for Longitudinal CSF Studies

Longitudinal glioma CSF acquisition via access devices placed at biopsy or resection is feasible and well tolerated. Additional takeaways and recommendations are presented in Table 1. We recommend for CSF to be obtained concurrently with standard-of-care MRIs. When a biopsy is clinically indicated, Ommaya reservoirs can be placed ventricularly while awaiting intra-operative pathology results, mitigating the impact of resection on monitoring or pharmacodynamic biomarker discovery. When placed at resection, longitudinal CSF from patients not immediately undergoing further treatment can be used to isolate resection-associated evolution of candidate biomarkers. Finally, control CSF samples should be acquired, including non-tumor controls such as multiple sclerosis or meningitis to filter out inflammatory markers, as well as malignant non-glioma controls to identify glioma-specific analytes. As a resource to the field to encourage longitudinal CSF studies, we have provided our IRB protocols and consent forms for Ommaya reservoir placement at the time of biopsy or resection, as well as a broad liquid biopsy biomarker protocol to acquire and bank samples from pre-existing CSF access devices longitudinally for research purposes (Supplementary materials).

## How and When to Incorporate CSF: Challenges and Opportunities

### Importance of Patient-Level Metadata and Guidelines: Sharing is Caring

CSF biomarker discovery is influenced by pre-analytical variables that have been reviewed excellently elsewhere,^19^ the impact of which varies across biomarker types. As longitudinal CSF is incorporated into multi-center trials and biobanking efforts, standardized approaches and step-by-step guidance for CSF biomarker studies should be developed by liquid biopsy consortia to increase the reliability of CSF as a biomarker source, which is currently ongoing by the liquid biopsy task force in the RANO group^22^ and the Brain-Liquid Biopsy Consortium.^86^ A robust infrastructure is needed to implement such protocols. While protocol standardization will take time, CSF biobanking efforts should not be deterred. Rather, we recommend meticulous documentation and publication of pre-analytical variables^19^ for each sample, as these can significantly impact biomarker discovery efforts (Supplemental Figure 1). Importantly, releasing only summary metadata for cohorts limits meaningful re-analysis across studies, as some patient-level covariates may not have been the studies’ primary endpoint. Thus, as this nascent field develops, patient-level data should also be shared while protecting patient identity for correlation to patient-level -omics data, including data related to anatomical, radiographical, and longitudinal variables that should be released with any -omics datasets. Data collection templates are provided in Supplemental materials. We also recommend for the liquid biopsy consortia and institutions to collaboratively prioritize and develop studies focused on testing the impact of such pre-analytical variables on key candidate biomarker types. Finally, biobanked CSF samples should be shared across institutions to accelerate biomarker discovery. Ultimately, prospective multi-institution studies collecting CSF at defined time points and working collectively through a consortium effort will be needed to validate glioma CSF biomarkers. However, this is currently hindered by material transfer agreements involving lengthy negotiations. Establishing pre-defined agreements within a consortium framework could streamline the process and promote collaboration.

### Cross-Validation Across Platforms and Institutions

CSF biomarker discovery is performed across institutional cores, research labs, and commercial platforms. Each may quantify and normalize different analytes using various methods. For example, multiple methods exist for the analysis of cell-free DNA, including but not limited to whole genome or exome sequencing, digital droplet PCR, BEAMing, targeted sequencing, and more,^21,87^ each of which have a different depth of sequencing that will impact mutation detection and the confidence in the studies’ results. Such variability across methods, particularly if methods are not fully reported, may in part explain differences in sensitivity and specificity across cfDNA-based studies in neuro-oncology. To allow for validation against published data, releasing both raw and normalized data open access is essential, utilizing standardized IDs from centralized databases to easily align analytes across public datasets. Moreover, assay performance can be influenced by variability in sample preparation quality and analyte isolation methodologies. Detailed protocols should be included in publications to enable independent validation and reproducibility. Of note, CLIA-certified assays will be needed for the clinical application of validated biomarkers.

### Incorporation with Other Specimens and Endpoints

#### Incorporation of CSF with Plasma/Serum Sampling

Plasma affords more routine availability for minimally invasive serial collection, while longitudinal CSF requires placement of a CSF access device or serial LPs. Nevertheless, CSF generally has improved sensitivity for glioma detection compared to plasma based on cfDNA genomic and methylomic analyses, due to less dilution and germline DNA contamination.^42,47,88,89^ However, one meta-analysis comparing serum versus CSF EVs suggested a similar sensitivity for the detection of GBM-associated EVs, with higher specificity in CSF.^90^ Moreover, another study identified IDHm mRNA from EVs in CSF, but not serum, from the same patients.^25^ For proteomics and metabolomics, identifying glioma-derived analytes in CSF is improved due to a higher signal-to-noise ratio than plasma. However, depending on the candidate biomarker, the tumor likely needs to have direct contact with CSF for some (e.g. cfDNA), but not all (e.g. D-2-HG) analytes. While 80% of recurrences occur around the resection cavity,^91–93^ gliomas are highly infiltrative and detecting distant progression is necessary. Accordingly, paired longitudinal plasma and CSF samples should ideally be obtained to identify biomarker types and scenarios where one source may outperform the other. Additionally, biomarker cross-validation in paired CSF and plasma can increase confidence regarding disease trajectory when results are concordant.

#### Incorporation of CSF with Tissue Sampling

Tissue remains the gold standard for disease diagnosis and monitoring in neuro-oncology^14^ and will be needed for CSF biomarker validation. However, serial biopsies are rarely ever performed, except in oncolytic virus studies of G47 Δ^13^ and CAN-3110^12^, and biopsies can be subject to sampling error^10,94^ as multiple regions are rarely sampled. To date, the concordance of GBM CSF cfDNA NGS with tissue has been reported to be around 40-50%.^43,54^ While serial tissues are infrequent, it is becoming increasingly accepted to perform a biopsy prior to resection for diagnostic confirmation,^14^ empowering routine comparison of valuable pre-versus-post-drug tissue samples on clinical trials. CSF collected at these same timepoints can be calibrated against tissue findings, and evaluated in post-operative serial CSF samples to assess the ongoing pharmacodynamic impact. Finally, multi-omic analyses across matched CSF, tissue, and/or tissue specimens, as recently performed with epigenomic and proteomic alterations in CSF for glioma diagnosis,^95^ can provide additional orthogonal evidence for identifying diagnostic and monitoring signatures in gliomas.

#### Incorporation of Different Multi-Omic Biomarkers Within CSF

As multi-omic glioma CSF studies evolve, new questions will arise regarding whether integrating different biomarker types could improve their performance.^95^ We have previously used D-2-HG levels to strengthen confidence in longitudinal IDH1 VAF changes.^54^ Moreover, cell free-methylated DNA immunoprecipitation sequencing has been used to create CSF methylomic classifiers reliably distinguishing gliomas from other diagnoses.^89,96^ This method leverages multiple biomarkers across the DNA methylome in a classification model, ensuring that glioma detection is based not on the presence of a single marker, but on a pattern of features across the methylome. A comparable strategy could be applied to integrate multi-omics CSF to construct models for accurate glioma detection, either at initial diagnosis or during monitoring. One pitfall of such models derived solely from CSF is that non-tumor variables, such as plasma-derived proteins, may risk being integrated into the feature set. To enrich for tumor-specific biomarkers, a second feature selection step can be performed to only include features present in tissue, toward improving the reliability and generalizability of robust tumor-specific biomarkers. As such, during model development, access to parallel tissue -omics data can refine CSF biomarker model performance.

#### Integration of CSF Biomarkers with Imaging, Survival, and in Clinical Practice

Biobanking longitudinal CSF samples enables multi-omic biomarker discovery and validation in glioma patients. Just as CSF can be correlated with findings in plasma and tissue, evaluating trajectories of different CSF biomarkers in a patient can enhance confidence in disease monitoring. In our experience, evaluating multiple tumor-specific analytes, such as D-2-HG and IDH1 VAF, can provide a “reality check” on the performance of each biomarker in real time, particularly when results are concordant.^54^ When results differ, further investigation is required to understand the discrepancies.

Standard imaging, including T1-Gad and T2-FLAIR sequences, is used to assess glioma treatment response according to RANO criteria.^2^ However, imaging alone is imperfectly sensitive due to treatment-related changes.^3^ Retrospective analyses of progression-free and overall survival can aid in defining disease trajectory but are limited in their real-time utility.^97^ Despite these limitations, MRIs and survival remain standard-of-care for assessing disease trajectory. When both MRIs and disease trajectory agree with one another, candidate CSF biomarkers can be benchmarked against these metrics. For broader adoption, including in community practice, CSF biomarkers can be integrated at key timepoints—such as early prognostic assessment or during equivocation for progression versus pseudoprogression.

Advanced imaging methods such as ^18^F-DOPA PET,^98^ diffusion-weighted imaging (DWI),^99,100^ and perfusion (dynamic susceptibility contrast)^101^ have not yet been incorporated into RANO in part due to the single-center, nonrandomized nature of prior studies. Nevertheless, these approaches may offer greater sensitivity for detecting gliomas compared to standard MRI^102^ and may provide more near-term endpoints on disease status compared to clinical endpoints like progression-free or overall survival. Few studies have correlated candidate glioma CSF biomarkers with advanced imaging, such as whether increased perfusion correlates with plasma-derived CSF proteins. Radiomics and radiogenomics are also under investigation as response assessment tools.^103^ Future research should evaluate whether integrating candidate CSF biomarkers with advanced imaging or radiomics models could enhance disease monitoring accuracy beyond what each modality achieves individually.

## Conclusions

CSF shows great potential as a multi-omic biomarker source for disease monitoring and pharmacodynamic evaluation in neuro-oncology. To discover and validate biomarkers rigorously, key factors must be considered, including CSF acquisition site, BBB disruption, and evolving surgical impacts across the CSF “time-space continuum.” Technical and safety considerations are important when longitudinal samples are obtained via access devices. Further efforts are needed to integrate CSF biomarker discovery with tissue and plasma sampling, advanced imaging, and traditional outcome measures like OS/PFS to robustly identify glioma-specific biomarkers. Improved sampling technologies and low-input assays may enhance biomarker studies. As multi-omics approaches to CSF biomarker discovery expand, artificial intelligence/machine learning could help integrate multiple biomarkers and correlate them with clinical outcomes. Finally, sharing samples and patient-level metadata with “-omics” results is essential to foster and accelerate communal progress in neuro-oncology biomarker efforts.

## Supplementary material

Supplementary material is available online at *Neuro-Oncology* (https://academic.oup.com/neuro-oncology).

noae276_suppl_Supplementary_Figure_S1

noae276_suppl_Supplementary_Materials

## Data Availability

All data in this review are available with request to the corresponding author or in the cited manuscripts.
