# Erratum to: A hitchhiker’s guide to cerebrospinal fluid biomarkers for neuro-oncology

**DOI:** 10.1093/neuonc/noaf148

**Published:** 2025-07-01

This is an erratum to: Cecile Riviere-Cazaux, Michael B Keough, Jeffrey A Zuccato, Rahul Kumar, Sebastian C Schulz, Arthur E Warrington, Michael W Ruff, Benjamin M Ellingson, Nader Sanai, Jian L Campian, Sani H Kizilbash, Ian F Parney, Gelareh Zadeh, Mustafa Khasraw, Tobias Kessler, Ugur Sener, Daniel P Cahill, Alireza Mansouri, Terry C Burns, A hitchhiker’s guide to cerebrospinal fluid biomarkers for neuro-oncology, *Neuro-Oncology*, 2024; https://doi.org/10.1093/neuonc/noae276

The following changes have been made to the originally published manuscript. The fifth co-author’s name is emended to read: “Sebastian C Schulz”

