# Supplementary material for: A hitchhiker’s guide to cerebrospinal fluid biomarkers for neuro-oncology
Source: Neuro Oncol. 2024 Dec 30;27(5):1165–79. doi: 10.1093/neuonc/noae276 (PMC12187377; doi:10.1093/neuonc/noae276)
Supplement: noae276_suppl_Supplementary_Figure_S1 [file noae276_suppl_supplementary_figure_s1.docx]

**Supplementary Materials**

**Supplementary Figure and Figure Legend**

**
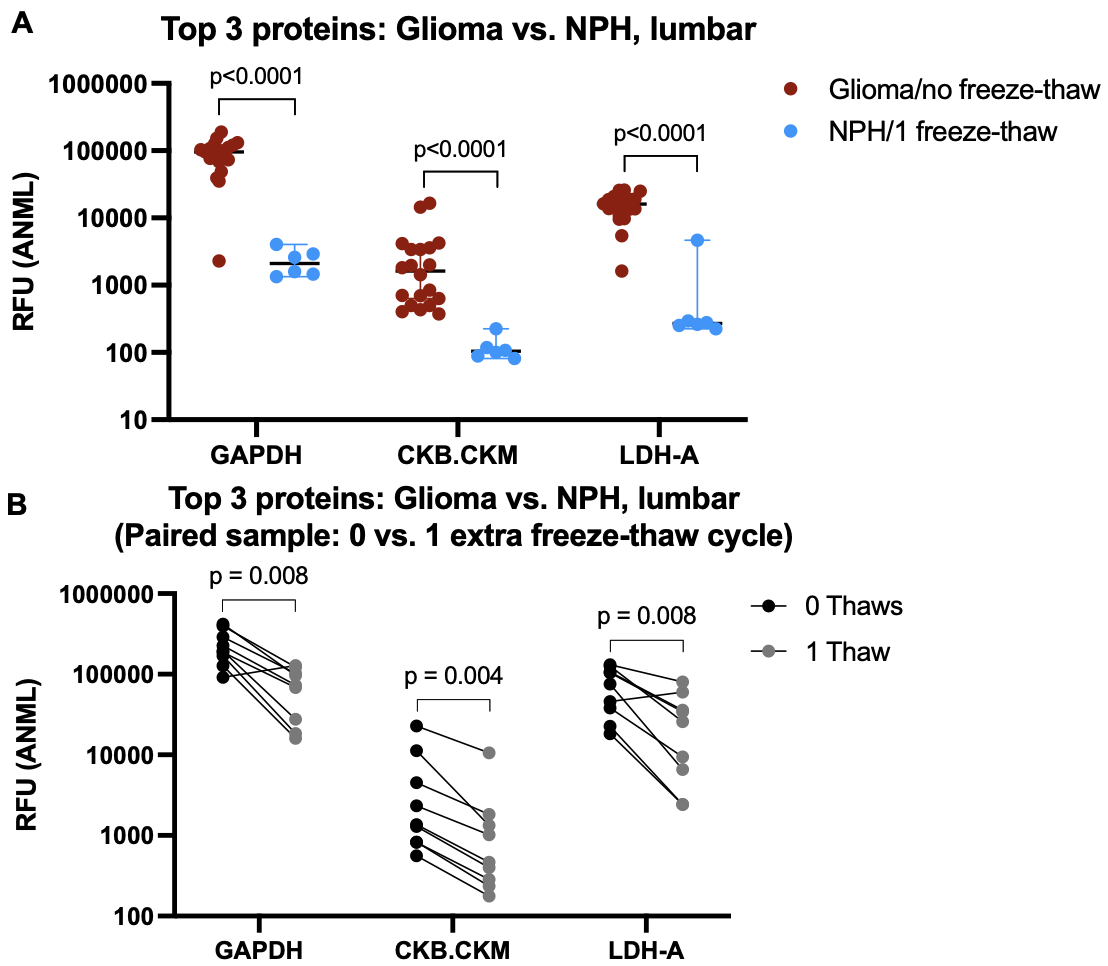
**

**Supplementary Figure 1. Freeze-thawing can adversely impact the discovery of glioma-derived biomarkers.**

**(A)** Lumbar cerebrospinal fluid (CSF) was compared between twenty patients with diffuse gliomas and six patients with normal pressure hydrocephalus (NPH). The NPH samples had been acquired from the Center for Multiple Sclerosis and Autoimmune Neurology at Mayo Clinic and had undergone one additional freeze-thaw cycle than the diffuse glioma samples. Mann-Whitney U-tests were performed to identify significantly different proteins between the lumbar glioma versus NPH samples; the top three significant proteins according to fold-change and p-value<0.05 were glyceraldehyde 3-phosphate dehydrogenase (GAPDH), creatine kinase M-type/creatine kinase B-type heterodimer (CKB.CKM) and lactate dehydrogenase A (LDH-A). **(B)** Nine pairs of CSF samples were evaluated: within each pair, the samples were identical other than one had undergone an extra freeze-thaw cycle. Wilcoxon signed-rank tests were then performed on the nine CSF pairs, demonstrating a significant decrease in detection of GAPDH, CKB/CKM, and LDH-A after freeze-thawing. As NPH samples underwent an extra freeze-thaw cycle relative to the glioma samples, the seemingly lower GAPDH, CKB.CKM, and LDH-A levels in (A) may have been confounded by the impact of freeze-thawing, highlighting the need for documentation of such pre-analytical variables.
