# Supplementary material for: A hitchhiker’s guide to cerebrospinal fluid biomarkers for neuro-oncology
Source: Neuro Oncol. 2024 Dec 30;27(5):1165–79. doi: 10.1093/neuonc/noae276 (PMC12187377; doi:10.1093/neuonc/noae276)
Supplement: noae276_suppl_Supplementary_Materials [file noae276_suppl_supplementary_materials.zip › noae276_suppl_materials/CSFreview_Lqbx_protocol_supp_final.docx]

**OVERVIEW OF PROTOCOL**

**Study title:** Liquid Biopsy Biomarkers for Brain Tumors

**Hypothesis:** Liquid biopsies, including cerebrospinal fluid (CSF), blood, and urine, are hypothesized to contain biomarkers that reflect disease burden, recurrence and response to therapy.

**Aims, purpose, or objectives:** To collect liquid biopsy specimens, including CSF, blood, and/or urine, from patients with brain tumors for future research.

**Background**: There remains no clinically available biomarker for any brain tumor. Imaging studies are frequently confounded by treatment-related artifacts. Biomarkers of disease burden and mechanistic biomarkers of response to therapy are urgently needed to empower advancements toward timely, effective and individualized patient care.

At present, most primary brain tumors are incurable. Despite potentially promising drugs under evaluation, translation has been hampered in part by challenges of CNS penetration and a repertoire of potential resistance mechanisms. There remains an urgent unmet need for reliable quantitative monitoring biomarkers that could accelerate translational progress toward curative therapies. Since the disease course for certain brain tumors can extend over several years, predictive or pharmacodynamic biomarkers that reflect tumor vulnerabilities or molecular impacts of therapy could accelerate progress toward new and individualized therapies. Toward this goal, we propose an avenue for CSF to be accessed from consenting patients to provide a resource for ongoing and future discovery efforts. Moreover, additional liquid biopsy specimens can be accessed longitudinally during the patient’s disease course for biomarker discovery, including blood and/or urine.

**Target accrual:** [insert number of patients for your institution]

**Subject population:** Single-institution series of adult patients with known or suspected brain tumor.

**Inclusion criteria:** Adult patients (≥ 18 years) with a known or suspected brain tumor who consent to a research-only procedure for CSF, blood, and/or urine collection/

**Exclusion criteria:** Members of a vulnerable population and/or unable to provide written informed consent will be excluded from participating in this study. Exception will be granted for patients with diminished capacity to consent if a legally authorized representative is available.

# Study objectives

## Primary objective

The primary objective will be to collect liquid biopsy samples that can provide a resource to the research community for biomarker discovery in patients with gliomas, including CSF, blood, and/or urine.

## Secondary objectives

## Secondary objectives include evaluating the feasibility of serial liquid biopsy sampling from patients with brain tumors for longitudinal evaluation of tumor biomarkers.

# BACKGROUND AND RATIONALE

## Background on brains tumors and biomarkers

## Despite initial sensitivity to chemotherapy and radiation, brain tumors frequently recur. Primary brain tumors (gliomas) inevitably become resistant to therapy and prove ultimately fatal. CSF is not routinely accessed for clinical purposes, but offers the highest concentration of biomarkers relevant to CNS disease, including brain tumors. Limited availability of CSF for research hampers efforts to identify biomarkers that could inform disease burden, therapeutic susceptibility, and response to therapy. Potentially informative biomarkers may include extracellular vesicles, metabolites, cell-free DNA, microRNAs, cytokines, and peptides, among others.

## Ultimately, relevant biomarkers may be identified and followed through peripheral blood. However, just as the blood brain barrier may hamper access of candidate drugs to the CNS, so can CNS biomarkers be hampered from access to serum. As such, CSF can provide more timely and sensitive access to certain CNS disease biomarkers, while providing reference samples for other studies seeking to identify less invasively accessible biomarkers. Additional liquid biopsy specimen types, like blood and urine, can also be accessed longitudinally, even in patients not undergoing CSF acquisition, each of which may also contain glioma biomarkers.

## Study rationale

## The goal of the study is to generate a repository of liquid biopsy samples relevant to neuro-oncology clinical research that may facilitate the identification of biomarkers predictive of disease burden, therapeutic susceptibility or response to therapy. This protocol will also provide a mechanism enabling liquid biopsy specimen access for patients or providers wishing to generate or evaluate patient-specific biomarkers as part of parallel efforts to develop individualized therapies. This study does not concern itself with the use of the samples obtained. However, it is expected that the samples will be maintained in the biorepository for the benefit of the research community and future patients who may benefit from resultant discoveries.

#

# 3 STUDY DESIGN/ SELECTION AND ENROLLMENT OF PARTICIPANTS

# The current protocol provides for CSF to be obtained via any percutaneously accessible CSF collection or reservoir (such as a VP shunt, Ommaya reservoir or pseudomeningocoele), implanted CSF access device (such as external ventricular drain, or lumbar drain) or lumbar puncture. Specifically, procedures to access CSF via these avenues may be performed under this IRB protocol for the sole purpose of research. Additionally, patients without brain tumors may have CSF access devices (i.e., for normal pressure hydrocephalus; after subarachnoid hemorrhage; after spinal surgery) where CSF is collected and then disposed of as medical waste. This protocol will also allow for the collection of CSF from such devices that were implanted for a clinical indication. As this CSF would have otherwise been disposed of, this poses no additional risk to these patients and allows for their samples to be used as controls to brain tumor samples.

# The entire central nervous system contains 150-250 mL of CSF, and is turned over continuously, with an average daily production of 500-700 mL; we will withdraw up to a maximum of 20 mL per patient per timepoint. The samples will be stored under the neuro-oncology biorepository. Clinically indicated procedures yielding CSF in excess of diagnostic needs may bank excess CSF through the neuro-oncology biorepository without enrollment in this protocol. To obtain other liquid biopsy specimen types, blood and/or urine may also be collected, including from patients who are not otherwise undergoing CSF collection. Patients may choose to undergo collection of one, two, or all three liquid biopsy specimen types.

# Since this protocol aims to provide a resource for identification of biomarkers to guide individualized therapies, patients may be requested to undergo liquid biopsy sampling during at least 2 time points. If one liquid biopsy sample was already obtained during a clinically indicated procedure, a single additional sample obtained through this protocol may be sufficient to identify biomarkers correlating with disease burden. Generally, timing of samples will be separated to reasonably anticipate at least a 20% change in tumor size between samples as a result of tumor resection, response to therapy, or tumor growth. The samples will be de-identified, processed and archived in the biorepository, after which samples will be available upon request to biorepository leadership for IRB-approved research studies.

**3.1 Subject population**

Single-institution series of patients with known or suspected brain tumor, who may be recruited at any point prior to, during, or following treatment. Additionally, patients without brain tumors who have CSF access devices or who are willing to undergo blood and/or urine collection may be recruited as controls. The goal will be to accrue [insert number of patients for your institution].

## 3.2 Inclusion criteria

Eligibility for study inclusion will be conveyed by any evidence of neoplasm involving the central nervous system (CNS) or its adjacent structures in contact with CSF. Such lesions may include but are not limited to intra-axial or extra-axial lesions, which could be benign, malignant or as yet undefined, involving the brain, spine, meninges, nerves, or vasculature or supporting structures. For CSF, eligibility for non-tumor control patients includes an already-existing clinically indicated CSF access device that is collecting/draining CSF. Blood or urine may be acquired from control subjects who do not have a CSF access device. Subjects must be 18 years of age or older.

**3.3 Exclusion criteria**

Patients who are under 18 years of age or are a member of a vulnerable population will be excluded from this study. Pregnant women or women who may be pregnant are specifically excluded from study participation. Exception will be granted for patients with diminished capacity to consent if a legally authorized representative is available. Patients without clinical or radiographic evidence of a potentially neoplastic CNS lesion will be excluded, unless they have an existing CSF access device that is draining CSF that would otherwise be disposed of as medical waste, or unless they are in the control cohort AND providing blood or urine samples Patients with an inability or unwillingness of individual or legal guardian/representative to give written informed consent will be excluded. Any patient for whom a clinical contraindication exists to the intended route of CSF access will be excluded from CSF collection; however, blood or urine samples may still be acquired. For example, a patient with a large posterior fossa mass lesion at risk of herniation, or a patient with coagulopathy, or other contraindication to lumbar puncture would not be eligible to participate via use of lumbar puncture for CSF access.

**3.4 Study enrollment procedures**

Patients seen in the hospital or clinic setting by Neurosurgery, Neuro Oncology, Oncology, Radiation Oncology, or Neurology at our institution who meet inclusion criteria and do not have exclusion criteria will be considered for enrollment the study. Patients will be provided with a layperson statement about the trial and asked if they would be interested in hearing about this research study. If they say yes, the MD will explain the study.

Patients are provided a consent form for this protocol along with an informed consent form for the biorepository and are provided an initial introduction to the study by the physician and/or appropriate study coordinator in the clinic or hospital setting. Both will be available to answer questions. The patient will be asked if they need more time and are welcome to take the consent for further review and self-reflection. There will be no set waiting period. The investigator will encourage the patient to take time to reach a decision about enrolling in the clinical trial. The patient is given the opportunity to ask questions about the informed consent document via telephone or at a subsequent office visit. If needed, the study coordinator will follow up after the initial visit to answer any questions that the patient may have, or to arrange for a follow-up visit with the investigator for further discussion prior to final decision regarding enrollment.

It is repeatedly emphasized in conversations between the patient, the study coordinator, and the investigator that participation in research is entirely voluntary, and that standard treatment options, or the option of no treatment, remain available to the patient. The risks and benefits of the investigational protocol are emphasized. This aspect of the conversation is also documented in the medical record. Phone numbers are provided in the consent document for independent parties who can discuss research protocols with the patient.

# STUDY INTERVENTIONS

## Interventions, administration, and duration

There are no medical treatments, drugs or novel devices planned for this study. Rather, sampling of CSF, blood, and/or urine is proposed through conventional collection mechanisms. Patients may be requested to undergo liquid biopsy sampling during at least two time points. Biomarkers will be evaluated for these time points. Timing of liquid biopsy samples will be separated to reasonably anticipate at least a 20% change in tumor size between samples as a result of tumor resection, response to therapy, or tumor growth. After liquid biopsy samples have been collected from two time points, subjects may elect to provide additional, optional collections, following the same time point guidelines. These optional collections would also be paid by the study and would occur during research-only visits. If subjects choose not to provide optional collections, they will remain in long-term follow-up for chart review until they have completed the study. For control patients, CSF will be obtained from their CSF access device before it would be disposed of as medical waste, i.e. bag attached to external ventricular drain or lumbar drain. Blood and/or urine may also be collected from these patients via conventional collection mechanisms.

**Baseline blood draw (Subjects >18 years of age):**

Participants will be given the option to donate a blood sample. If they agree, up to three 10 mL EDTA tubes will be collected at the visit 1 day 0 to generate up to 9 1 mL aliquots of plasma, 1 aliquot of WBCs for genomic DNA, and three aliquots of buffy coat (subject to change based on biobank studies). Blood is used as a matched normal sample for cell free DNA and other biomarker analysis or if a patient does not have a tumor, as a normal control.

**Subsequent blood draw (subjects >18 years of age):**

Total volume of follow-up blood draws will not exceed 50mL in a single day and not more than 110 mL in any 4-week interval. Sampling may be divided across multiple timepoints if associated with co-enrolled investigational interventions. At any one visit, up to three 10 mL EDTA tubes will be collected to generate up to 9 1 mL aliquots of plasma, 1 aliquot of WBCs for genomic DNA, and three aliquots of buffy coat (subject to change based on biobank studies).

**Urine collection:**

A midstream urine sample should be collected by having the patient void the first part of the urine into the toilet, then bringing the urine collection cup into the “midstream” to collect the urine sample (up to 10 mL). The remainder of the urine may be voided directly into the toilet. No part of the interior of the collection cup should be touched with the hands or any other part of the body.

## Handling of study interventions

## The CSF samples will be obtained via lumbar puncture, percutaneous access to a CSF reservoir or collection, or access to an external CSF access device. Blood and/or urine will be obtained as in section 4.1. Up to 20 mL CSF will be withdrawn from each patient at a single timepoint. The samples will be kept on ice for transport, and then processed by the neuro-oncology biorepository personnel. The informed consent form for the neuro-oncology biorepository specifically addresses details of the banking process, confidentiality and use of samples inside and outside of the institution.

This protocol is intended to facilitate both biomarker discovery and biobanking efforts. Although no specific laboratory studies are mandated with the samples collected, the protocol shall provide a vehicle through which pertinent research results may be returned to interested and consented patients. This may occur if and when the following three criteria are met: (i) a biomarker result is generated based on a well-established assay approximating the expected reliability of a CLIA assay, or a research assay in development toward a CLIA assay, (ii) results are deemed by the PI to be of potential relevance to the patient’s clinical management, and (iii) the patient elected on their consent form to be notified if and when such results became available. In such cases, patients will be provided with the option to receive this information. If they agree, the result will be provided verbally or in writing, and the research result, provisional reference range and other pertinent information will be result documented in a research note within the patient’s medical record.

## Concomitant interventions

## There are no restricted or mandated interventions or clinical management directions related to this trial. Patients will receive conventional, standard of care medical and diagnostic evaluations, treatments – including chemotherapy and radiation, interventions – including surgical treatment, and routine follow up. No procedure, laboratory or other costs incurred as part of liquid biopsy access, sample analysis or sample banking will be billed to the patient or the patient’s insurance.

## Adherence assessment

Patients will be expected to adhere to standard of care follow up and communication regarding their clinical course. There are no requirements of the patient either prior to, during, or following participating in the trial. It is not expected that patients would require additional medical care or medications because of the trial, though conceivable risks will be detailed. Should any complications occur, they will be they will be managed via standard protocols and billed through insurance as would be the case for any other patient.

# STUDY PROCEDURES

## Schedule of evaluations

| ***Assessment*** | ***Screening: Visit (Day-14 to Day -1)*** | ***Sample Collection Visit 1 (Day 0)*** | ***Second Collection Visit 2 (if applicable)***  ***(Day 0 + TBD)*** | ***Additional Collections (optional)*** |
| --- | --- | --- | --- | --- |
| Informed Consent Form | **X** |  |  |  |
| Inclusion/Exclusion Criteria | **X** |  |  |  |
| Liquid biopsy Collection |  | **X** | **X** | **X** |
| Survey for Adverse Events* |  | **X** | **X** | **X** |
| Blood Collection |  | **X** |  |  |

**Only for patients undergoing CSF collection directly from a CSF access device; no adverse event evaluation required for patients undergoing blood/urine collection, or for patients where CSF is obtained as medical waste from an EVD or lumbar drain bag.*

# SAFETY ASSESSMENTS

## Specification of safety parameters

Patients will undergo specific counseling to discuss possible signs of infection, CSF leak, or shunt malfunction (if applicable) that should prompt consultation with a medical provider. When indicated, patients will undergo standard evaluations for any of these concerns, which may or may not include laboratory testing, vital exam, surgical or procedural site inspection, neurologic examination, and imaging (x-ray, CT, MRI), if medically indicated.

## Risks of participation

Participation in this clinical trial entails risks. These risks are similar to those experienced during routine care a patient may receive as a part of their medical surveillance and treatment. The clinical study requires obtaining CSF for biomarker analysis. This may be accomplished via access to any percutaneously accessible CSF collection (such as a pseudomeningocoele), implanted device with an appropriately accessible reservoir (such as VP shunt, or Ommaya reservoir), or external CSF access device (such as external ventricular drain, or lumbar drain), and/or via lumbar puncture. Such procedures are often performed in the clinic setting, emergency department room, or general care floor hospital room. None of these CSF access approaches require sedation or IV medications. Local anesthesia is utilized for lumbar puncture, creating a minimal risk for a local immunologic reaction. There is a small risk of local site hemorrhage related to the shunt tap needle (usually 25 gauge) or LP needle, which is controlled with brief local pressure. During the LP, there is risk of nerve root irritation or injury—usually manifested by a short, acute radicular pain that resolves when the stimulus is removed. The lack of systemic sedation would facilitate rapid identification of nerve root irritation that can be promptly rectified during the procedure by redirecting the needle. There is a small, but important risk of infection from accessing the CSF space. All procedures are performed with meticulous local skin preparation using and standardized sterile technique to minimize risk. Oral or IV medications are not required for the procedure, although may be indicated if a patient experiences subsequent signs or symptoms of an infection. For an implanted device, if an infection is suspected, normal diagnostic studies and interventions would be undertaken – including repeat shunt tap for laboratory studies, possible antibiotics, and with the presence of an infected device – possible exploration and/or replacement. The risk of infection following LP is expected to be well under 1% (Baer ET. *Post-dural puncture bacterial meningitis*. Anesthesiology 2006; 105(2):381; Evans RW. *Complications of Lumbar Puncture*. Neurologic Clinics. 1998(16)1:83-105). Similarly, the risk of an infection following VP shunt or Ommaya reservoir access is very low – with documented rate of infections being under 0.1% (Spiegelman L, et al. *What is the risk of infecting a cerebrospinal fluid-diverting shunt with percutaneous tapping?* JNS Pediatrics 2014; 14:336-339). Finally, LP may result in temporary post-procedural postural headache that often resolves with hydration, caffeine, and rest within a few days. In rare remote cases, persistence may require blood patching with an interventional radiologist. These risks are cumulatively quite low and are reasonable to facilitate progress toward identification of biomarkers and insights that may improve standards of care for patients with brain tumors.

For control patients without tumors who have CSF access devices, this study poses no risk, as we are simply collecting the CSF from the bag that would have otherwise been disposed of as medical waste.

There are no risks associated with urine collection. The only risk associated with blood sampling is minor discomfort with venipuncture and potentially bruising at the needle insertion site, although this is not common and is expected to quickly resolve.

## Adverse events and serious Adverse Events

At each contact with the subject, the study team will seek information on AEs by specific questioning and, as appropriate, by examination. All AEs occurring during the study period will be recorded. The clinical course of each event will be followed until resolution, stabilization, or until it has been ultimately determined that the study treatment or participation is not the probable cause.

Attribution of adverse events to the study, either directly or indirectly, would require evaluation of clinical context. For instance, after surgical resection of a brain lesion, the white blood cell counts naturally rises – in part due to the surgery itself and in part due to a dexamethasone taper that all patients received. As such, a device tap occurring in proximity to a surgical procedure or steroid administration could lead to some abnormal laboratory values concerning for possible infection. Definitive evidence of infection would require laboratory and culture evaluation of CSF. This would be obtained when clinically indicated in the context of clinical signs and symptoms including vital signs, neurological exam, systemic laboratory values (ESR, CRP), and possibly imaging studies. Similarly, clinical evaluation and imaging studies will be used to arrive at a diagnosis of intracranial hypotension leading to positional headaches which may very rarely complicate a lumbar puncture.

***Adverse Event (AE):***

As a part of conventional follow up, patients are routinely assessed with questions meant to address for development of adverse medical problems – including “any changes in neurologic function”, “any fevers, night sweats, shaking chills”, “wound breakdown or drainage”, “new or worsening headaches”, “headache brought on by standing or rising to an upright position”, etc. Answers to these questions will be formally addressed and documented in the medical record for each virtual and/or in-person encounter. The PI will evaluate the event and determine the necessary follow-up and reporting required.

## Reporting Procedures

Internal Data and Safety Monitoring Board

The neurosurgery Data Safety and Monitoring Board will be utilized to review the study documents and severe AEs related to participation in this trial.

*6.4.1 Severity of Event*

• Mild – Events require minimal or no treatment and do not interfere with the participant’s daily activities. This may include temporarily mild headache that does not require treatment or hospitalization, non-infectious wound inflammatory reaction that does not require medication or intervention, or self-limiting orthostatic headache.

• Moderate – Events result in a low level of inconvenience or concern with the therapeutic measures. Moderate events may cause some interference with functioning. This may include concern for shunt or LP site infection that requires prophylactic oral antibiotics, but not IV antibiotics or procedural intervention. Orthostatic headache that requires conservative, symptomatic measures, but no imaging or treatment measures; or orthostatic headache that requires a one-time blood patch treatment from interventional radiology.

• Severe – Events interrupt a participant’s usual daily activity and may require systemic drug therapy or other treatment. Severe events are usually potentially life-threatening or incapacitating. Of note, the term “severe” does not necessarily equate to “serious”. This may include surgical exploration of a VP shunt, and possible re-implantation of a new system, along with IV antibiotics.

**6.5 Relationship to study intervention**

All adverse events (AEs) must have their relationship to study intervention assessed by a qualified medical professional who evaluates the available documentation and/or examines the patient and reaches a conclusion based on temporal relationship and his/her clinical judgment. The degree of certainty about causality will be graded using the categories below. The DSMB will assess all ambiguity. Any enrolled participant deemed to have a “definitely related” event will not undergo further specimen collection, and would continue to receive standard of care. For “possibly related” events, a formal assessment would take place among the PI and co-investigators to provide clinical context and reach a consensus regarding whether the participant should be removed from further study collections. If there are mixed conclusions, the patient would be reviewed by the DSMB. For “not related” deemed events, the participant would continue on the trial as planned unless their situation mandates withdrawal for any reason.

• Definitely Related – There is clear evidence to suggest a causal relationship, and other possible contributing factors can be ruled out. The clinical event, including an abnormal laboratory test result, occurs in a plausible time relationship to sample acquisition and cannot be explained by concurrent disease or other drugs or chemicals.

• Possibly Related – There is some evidence to suggest a causal relationship. However, other factors may have contributed to the event (e.g., the participant’s clinical condition, other concomitant events). Although an AE may rate only as “possibly related” soon after discovery, it can be flagged as requiring more information and later be upgraded to “probably related” or “definitely related”, as appropriate.

• Not Related – The AE is completely independent of study intervention administration, and/or evidence exists that the event is definitely related to another etiology. There must be an alternative, definitive etiology documented by a qualified medical professional.

*6.5.1 Follow-up for Adverse Events*

Patient will receive clinical assessment at 30 days (+14) following each sample collection, which may coincide with Neuro-Oncologic follow-up. This may also take place over the telephone or virtually, depending on patient preference. The patient is additionally requested to call to report any evidence of potential AEs if noted outside of scheduled follow-up.

All AEs including local and systemic reactions not meeting the criteria for severe AEs (SAE) will be documented in the medical record. Information to be collected includes event description, time of onset, qualified medical professional’s assessment of severity, relationship to study procedure (assessed only by those with the training and authority to make a diagnosis), and time of resolution/stabilization of the event. Patients with brain tumors are unfortunately expected in most cases to experience tumor progression and subsequent neurological decline. However, any infection of any implanted device at any time will be documented. Chart review will be performed at 1 year for such patients to screen for any potentially insidious infection that may have become clinically manifest in the prior year. All AEs will be followed to adequate resolution.

Changes in the severity of an AE will be documented to allow an assessment of the duration of the event at each level of severity to be performed.

The PI will record all reportable events with start dates occurring any time after informed consent, including screening for CNS infection up to 1 year after the final sample collection.

# INTERVENTION DISCONTINUATION

Subjects may withdraw voluntarily from participation in the study at any time and for any reason. Subjects that experience “Directly Related” or mixed consensus “Possibly Related” AEs or SAEs will be discontinued from further specimen collection.

# STATISTICAL CONSIDERATIONS

## General design issues

This project revolves around biomarker collection and banking. No analysis of samples is required as part of this trial. However, should analysis be performed, this trial provides a vehicle for such results to be evaluated for potential clinical relevance.

## Primary objective

The primary objective will be to collect liquid biopsy samples that can provide a resource to the research community for biomarker discovery.

## Secondary objectives

Secondary objectives include evaluating the feasibility of serial liquid biopsy sampling from patients with brain tumors for longitudinal evaluation of tumor biomarkers.

## Interim analyses and stopping rules

An interim analysis is planned following 15 patients have been enrolled – 15% of planned enrollment. The study will undergo preliminary assessment of safety, to encompass the number of SAEs overall, the number of occurrences of a particular type of SAE, reactions, or increased frequency of events. Such findings are presented to the DSMB.

# DATA COLLECTION AND QUALITY ASSURANCE

## Data collection and management

Patient data will be prospectively collected and managed in a secure Excel database that will only be available to individuals approved for IRB participation. The patient samples will receive a code to identify biobanked specimens for appropriate reference. If the patient elects to receive the results of research studies deemed potentially relevant to their care, the research result will be documented in a research note in the patient’s medical record.

## Quality Assurance

### *9.2.1 Protocol Deviations*

Protocol deviations will be logged and reported at continuing review. If the deviation is related to an AE/SAE, it will be reported in accordance with AE/SAE regulations and subsequently reported at continuing review.

### *9.2.2 Monitoring*

Site monitoring by the departmental Research Counsel will be completed for assuring protocol compliance and data quality at the clinical sites, including review of records, consent forms, etc. The PI is responsible for material review and the schedule for reviews, which will also be performed independently DSMB.

# PARTICIPANT RIGHTS AND CONFIDENTIALITY

## Institutional Review Board (IRB) Review

This protocol and the informed consent document and any subsequent modifications will be reviewed and approved by the IRB.

## Informed consent form

The Informed Consent Forms (ICF) will be Institutional Review Board (IRB)-approved, and the participant will be asked to read and review the document. The investigator will explain the research study to the participant and answer any questions that may arise. A verbal explanation will be provided in terms suited to the participant’s comprehension of the purposes, procedures, and potential risks of the study and of their rights as research participants. Participants will be informed that participation is voluntary and that they may withdraw from the study at any time, without prejudice, and that the quality of their medical care will not be adversely affected if they decline to participate in this study. Participants will have the opportunity to carefully review the written consent form and ask questions prior to signing. The participants will be given a copy of the ICF so that they may discuss the study with their family or surrogates or think about it prior to agreeing to participate. The informed consent process will be conducted and documented in the source document (including the date), and the form signed, before the participant undergoes any study-specific procedures. A copy of the signed informed consent document will be given to the participants for their records.

## Participant confidentiality

Any data, specimens, forms, or other records will be identified only by a participant identification number (Participant ID, PID) to maintain confidentiality. All records will be kept in a locked file cabinet or on a secure password-protected institutional computer or server. Since banked samples will be maintained through the neuro-oncology biorepository, all confidentiality processes and procedures will be used in association with the samples acquired on this protocol.

## Study discontinuation

The study may be discontinued at any time by the IRB or DSMB as part of their duties to ensure that research participants are protected.

# ETHICAL CONSIDERATIONS

This study will be conducted in accordance with the Declaration of Helsinki, CIOMS, and International Ethical Guidelines for Biomedical Research Involving Human Subjects (2002).

# COMMITTEES

The departmental research committee will provide initial study review prior to IRB submission. The Rochester neurosurgery DSMB will conduct periodic assessment for safety and study coordination. This study will be reviewed and approved by the Institutional Review Board prior to patient enrollment or data collection.

# PUBLICATION OF RESEARCH FINDINGS

Publication of the results of this trial will be governed by the policies and procedures developed in collaboration with the PI, Neuro-oncology biorepository, and IRB.

# References

1. Xiong, N. *et al.* Using arterial–venous analysis to characterize cancer metabolic consumption in patients. *Nature Communications* **11**, 3169, doi:10.1038/s41467-020-16810-8 (2020).
2. Baer ET. *Post-dural puncture bacterial meningitis*. Anesthesiology 2006; 105(2):381
3. Evans RW. *Complications of Lumbar Puncture*. Neurologic Clinics. 1998(16)1:83-105).
4. Spiegelman L, Asija R, Da Silva SL, et al. *What is the risk of infecting a cerebrospinal fluid-diverting shunt with percutaneous tapping?* JNS Pediatrics 2014; 14:336-339
