# Supplementary material for: A hitchhiker’s guide to cerebrospinal fluid biomarkers for neuro-oncology
Source: Neuro Oncol. 2024 Dec 30;27(5):1165–79. doi: 10.1093/neuonc/noae276 (PMC12187377; doi:10.1093/neuonc/noae276)
Supplement: noae276_suppl_Supplementary_Materials [file noae276_suppl_supplementary_materials.zip › noae276_suppl_materials/CSFreview_Ommaya-at-bx_protocol_supp_final.docx]

Ommaya Reservoir Placement at the Time of Biopsy for Longitudinal Biomarker Collection

**Table of Contents**

Study Summary 4

1 Introduction 5

1.1 Background 5

1.2 Study rationale 5

1.2.1 Anticipated risks 6

1.2.2 Potential benefits 6

1.3 Anticipated duration of the clinical Investigation 7

2 Study Objectives 7

3 Study Design 7

3.1 General design 7

3.2 Primary endpoint 7

3.3 Secondary endpoint 7

4 Subject Selection, Enrollment and Withdrawal 8

4.1 Inclusion criteria 8

4.2 Exclusion criteria 8

4.3 Subject recruitment, enrollment and screening 8

4.4 Early Withdrawal of Subjects 8

4.4.1 When and how to withdraw subjects 8

4.4.2 Data collection and follow-up for withdrawn subjects 9

5 Ommaya description 9

5.1 Description 9

5.2 Preparation and administration/implantation of Ommaya reservoir 9

5.3 Prior and concomitant therapy 9

5.4 Packaging and labeling 9

5.5 Receiving, storage, distribution and return 9

5.5.1 Device acquisition 9

5.5.2 Storage 9

5.5.3 Distribution of study device 10

6 Study Procedures 10

7 Statistical Plan 13

7.1 Sample size determination 13

7.2 Statistical methods 13

7.3 Subject population(s) for analysis 13

8 Safety and Adverse Events 13

8.1 Definitions 13

8.2 Recording of adverse events 15

8.3 Reporting of unanticipated adverse device effects and unanticipated problems 17

8.3.1 Investigator reporting, notifying IRB 18

8.4 Stopping Rules 18

8.5 Medical Monitoring 18

8.5.1 Internal Data and Safety Monitoring Board 18

9 Data Handling and Record Keeping 19

9.1 Confidentiality 19

9.2 Source documents 19

9.3 Case Report Forms 19

9.4 Records Retention 20

10 Study Monitoring, Auditing, and Inspecting 20

10.1 Study Monitoring Plan 20

10.2 Auditing and Inspecting 21

11 Ethical Considerations 21

12 Study Finances 21

12.1 Funding Source 21

12.2 Conflict of Interest 21

13 Publication Plan 21

14 References 22

List of Abbreviations

| AE | Adverse Event/Adverse Experience |
| --- | --- |
| CFR | Code of Federal Regulations |
| CRF | Case Report Form |
| DSMB | Data and Safety Monitoring Board |
| GCP | Good Clinical Practice |
| HIPAA | Health Insurance Portability and Accountability Act |
| IDE | Investigational Device Exemption |
| IRB | Institutional Review Board |
| PHI | Protected Health Information |
| PI | Principal Investigator |
| SAE | Serious Adverse Event/Serious Adverse Experience |
| SOP | Standard Operating Procedure |
| UADE | Unanticipated Adverse Device Effect |

# Study Summary

| Title | Ommaya Reservoir Placement at the time of Biopsy for Longitudinal Biomarker Collection |
| --- | --- |
| Running Title | Ommaya at biopsy |
| IRB Protocol Number | TBD |
| Phase | N/A |
| Overall Study Duration | 2 years |
| Subject Participation Duration | Dependent on patient and disease course. |
| Objectives | 1. To determine the feasibility of placing Ommaya reservoirs at the time of biopsy to enable longitudinal CSF monitoring.  2. To biobank cerebrospinal fluid throughout a patient’s disease course and treatment. |
| Number of Subjects | 10 |
| Diagnosis and Main Inclusion Criteria | Diagnosis: brain tumor (primary or metastatic)  Main inclusion criteria:   - Age > 18 years. - Clinical and radiographic evidence suggesting a diagnosis of a brain tumor. - Planned biopsy for suspected or previously diagnosed brain tumor as part of routine clinical care at the institution. - Willingness of the patient or an authorized representative to provide informed consent - Patient is willing to have their Ommaya sampled on at least 2 future occasions. - Patients is willing to have CSF banked through the neuro-oncology biorepository (requires a separate signature) |
| Devices utilized in study | Ommaya reservoir and ventricular catheter |
| Duration of Exposure | Continuous, from time of implantation |
| Statistical Methodology | Feasibility – biomarker discovery from cerebrospinal fluid, including, but not limited to, metabolomics, proteomics, sequencing/methylation from cell-free DNA, extracellular vesicles, etc. |

# Introduction

This document is a protocol for a human research study. This study will be carried out in accordance with the procedures described in this protocol, applicable United States government regulations and institutional policies and procedures.

## Background

Brain tumors consist of primary and metastatic lesions, the former of which includes gliomas. The most aggressive type of glioma is glioblastoma, which is the most common and fatal primary brain tumor, with a median survival of only 15 months^1^. While low-grade gliomas carry a better prognosis, they are the most common malignant primary CNS tumor in younger patients and inevitably progress to higher, more aggressive grades^2-5^. Moreover, low-grade gliomas can induce neurological morbidity^6^. No drug has improved survival for patients with high-grade gliomas since temozolomide in 2005^1^. Recent evidence suggests that IDH inhibitors, such as vorasidenib, may delay the time to progression after resection of IDH-mutant gliomas but are not curative^7^. Preclinical research relies on models that imperfectly reflect each patient’s tumor. Additionally, few methods exist to longitudinally evaluate each patient’s brain tumor, in large part due to the relative inaccessibility of their lesion after surgery. As such, there is a significant need for methods that can be utilized to gain clinical and biologically-relevant insights into each patient’s brain tumor for disease monitoring and identification of therapeutic vulnerabilities throughout their disease course.

Biomarkers may facilitate disease monitoring and therapeutic response assessment^8^. However, plasma-derived biomarkers for brain tumors have had limited success to date, perhaps due to the limitations imposed by the blood-brain barrier. In contrast, cerebrospinal fluid (CSF) is emerging as a robust source of tumor-derived biomarkers^9^. While lumbar punctures can be performed serially, these are uncomfortable for patients, decreasing the practical availability of serial CSF samples. Furthermore, we have preliminary found that lumbar CSF is not as rich of a source of tumor-derived biomarkers as intracranial CSF. CSF access devices, such as Ommaya reservoirs^10^, can be utilized to longitudinally access intracranial CSF.

Ommaya reservoirs were introduced in 1963 to allow access to intraventricular CSF^10^. Since then, reservoirs have been utilized to deliver intrathecal chemotherapies and antibiotics, in addition to allow for aspiration of extra CSF and cysts^11^. Multiple clinical trials have utilized Ommaya reservoirs for longitudinal CSF access and numerous retrospective studies have demonstrated their safety^12-17^. As such, longitudinal CSF access via Ommaya reservoirs placed at the time of biopsy could be utilized for discovery of novel biomarkers indicative of disease state and therapeutic response in patients with brain tumors.

## Study rationale

Longitudinal CSF is emerging as an abundant source of biomarkers that could be utilized for disease monitoring and therapeutic response assessment. While access to longitudinal intracranial CSF has been limited in the past, use of Ommaya reservoirs from the time of biopsy throughout a patient’s disease course can overcome this barrier, providing an abundant source of analytes for biomarker discovery and validation. We currently have an open protocol for placement of Ommaya reservoirs during surgical resection. However, many patient’s tumors are not amenable to safe surgical resection. Such patients may be at highest risk of rapid recurrence and in greatest need of translationally relevant insights that could be generated from longitudinal CSF monitoring. This protocol will enable longitudinal acquisition of intracranial CSF samples from the time of initial diagnosis at biopsy throughout disease evolution and therapeutic interventions toward our goal of biomarker discovery and validation during standard of care and/or experimental therapies.

### Anticipated risks

One of the main risks associated with Ommaya reservoir placement and CSF extraction is infections. A retrospective series of 501 patients specifically evaluating Ommaya reservoir related infections reported a reservoir-related infection rate of 8%; other studies report incidences ranging from 3% to 13% when placed for intrathecal delivery in patients with brain tumors^18,19^. Symptoms could include mild to moderate fever and nuchal rigidity. More serious symptoms from infections such obnubilations, seizures or systemic affection, under the context of Ommaya reservoir tapping, are rare^18^. To minimize these risks, a specific protocol including use of Bactiseal-impregnated catheters and antibiotics, is utilized at the time of Ommaya reservoir placement, as described in section 6. Patients will also be monitored post-operatively per standard of care, with careful attention toward any potential signs of infection. Patients may also receive post-operative Bactrim to further minimize the risk of infection. Clinical judgement will be deployed to select patients for the trial who are not at unreasonably high risk of wound infection. However, infection is an established risk of Ommaya reservoir placement and will be monitored accordingly.

Other complications associated with Ommaya reservoir placement are similar to those of placing an external ventricular drain, including hemorrhage (1-3%, most often if thrombocytopenic) or injury to adjacent areas from catheter misplacement. All catheters will be placed under neuro-navigation guidance with trajectories to the ventricle that minimize the risk to nearby structures. We do not expect the risk of neurological deficit will be significantly increased by leaving an Ommaya reservoir at the time of biopsy. Nevertheless, neurological deficit occurring after surgery will be monitored and individually assessed for the possibility of attribution.

If a patient has a lesion where there is uncertainty as to whether it is or is not a malignancy of any type, the surgical team will wait for preliminary pathology results before placing an Ommaya. Waiting for preliminary pathology results may extend the time under anesthesia for approximately 20 minutes.

### Potential benefits

Availability of an Ommaya reservoir may enable the patient’s providers to access CSF to address questions of clinical relevance to help understand disease burden and trajectory. For example, CSF can be leveraged for an increasing number of assays through CLIA laboratories, spanning cytology, protein level, metabolite abundance (including D-2-HG for patients with IDH-mutant gliomas), and cell-free DNA. Research results obtained in a CLIA environment may be reported to patients and providers within a research note, even though they may not be reported as clinical results. It is hoped that participation in this trial will enable new discoveries that may be of benefit to the field and to future patients. Ultimately, it is our goal to identify and validate biomarkers of therapeutic response that could be utilized to more accurately determine if a current treatment is efficacious, minimizing the time spent on ineffective therapies, or even providing insights regarding optimal future therapies. Indeed, clinical trials are currently under development in our group that leverage results obtained from longitudinal CSF samples to guide therapeutic decision-making in an individualized manner. It is therefore plausible that the potential benefits of having an Ommaya reservoir may increase during the lifetime of the patient.

## Anticipated duration of the clinical Investigation

The duration of the clinical investigation will be dependent on the patient’s disease course. If placed in a patient with a low-grade glioma at the time of biopsy, follow-up could be as long as ten years; if placed in a patient with a high-grade glioma, study duration may be closer to fifteen months.

# Study Objectives

**2.1 Primary objective**

To determine the feasibility of placing Ommaya reservoirs at the time of biopsy to enable longitudinal CSF monitoring.

**2.2 Exploratory objective**

To biobank cerebrospinal fluid throughout a patient’s disease course and treatment.

# Study Design

## General design

This is a pilot study aimed at obtaining CSF samples for biomarker-based assays and biobanking throughout a patient’s disease course from the time of diagnosis at biopsy.

## Primary endpoint

Feasibility will be assessed based on the percent of patients for whom an Ommaya reservoir is successfully placed at the time of tissue biopsy without attributable complication.

## Secondary endpoint

Utility will be assessed based on the percent of patients for whom multiple (2 or more) CSF samples are successfully obtained as a result of their participation in this protocol.

# Subject Selection, Enrollment and Withdrawal

## Inclusion criteria

- Age > 18 years.
- Clinical and radiographic evidence suggesting a diagnosis of a brain tumor.
- Planned biopsy for suspected or previously diagnosed brain tumor as part of routine clinical care at the institution.
- Willingness of the patient to provide informed consent.
- Patient is willing to have their Ommaya sampled on at least 2 future occasions.
- Patients is willing to have CSF banked through the neuro-oncology biorepository (requires a separate signature).

## Exclusion criteria

- Vulnerable populations including pregnant women, prisoners, and individuals <18 years old.
- Patients who are not appropriate candidates for biopsy due to current or past medical history or uncontrolled current illness.
- Prior history of any wound infection.
- Any patient who the surgeon feels is not an optimal candidate for Ommaya reservoir placement. Such reasons may include, but will not be limited to, surgical anatomy, clinical evidence of immunosuppression, and/or elevated risk of wound infection due to diabetes, smoking history, morbid obesity, or any other concerns.

## Subject recruitment, enrollment and screening

Patients will be recruited from the neuro-oncology and neurosurgery clinics who require biopsy for a suspected primary or recurrent brain tumor. Patients will provide informed consent for Ommaya reservoir placement prior to proceeding to surgery for brain biopsy.

## Early Withdrawal of Subjects

### When and how to withdraw subjects

Subjects may withdraw at any time for any reason by verbal notification to any member of the surgical or study team. The surgeon may elect to not place the Ommaya reservoir during the surgery for any reason, including if optimal surgical access for placement of the Ommaya reservoir is not available, or if the surgeon feels completion of the Ommaya reservoir would unduly delay, prolong, or otherwise interfere with the surgery. Subjects who withdraw or are withdrawn after placement or attempted placement of the Ommaya reservoir will continue to be followed for collection of any data related to potential adverse events. Subjects that are withdrawn from the study at any point can be replaced toward the total accrual goal.

### Data collection and follow-up for withdrawn subjects

Subjects who withdraw or are withdrawn after placement or attempted placement of an Ommaya reservoir will continue to be followed for collection of any data related to potential adverse events.

# Ommaya description

## Description

The Ommaya reservoir is a subcutaneous CSF access device that is connected to a ventricular catheter. The ventricular catheter is inserted under stereotactic guidance into the ventricles in order to provide longitudinal access to CSF. This system will be utilized in patients with suspected brain tumors who are undergoing a clinically indicated biopsy. The most common Ommaya reservoirs typically utilized by our teams are the Natus Side- or bottom-inlet CSF reservoir (1.5 cm), NT8501214). The ventricular catheter most commonly utilized is the Integra Codman® Bactiseal ® ventricular catheter. Products may be modified depending on their availability, but this is not expected to impact study outcomes or patient safety. Serial numbers and lot/batch numbers are tracked for each device using a case report form and device accountability form.

## Preparation and administration/implantation of Ommaya reservoir

Please see section 6 for instructions on implantation of the Ommaya reservoir and its ventricular catheter. Ommayas and catheters are maintained in their sterile packaging until immediately prior to device utilization. No further preparation is needed.

## Prior and concomitant therapy

Prior and concomitant therapy information may be evaluated, but will not typically exclude patients from the study, except at the surgeon or PI’s discretion. Per standard neurosurgical procedure, anticoagulants must be held prior to biopsy to minimize the risk of hemorrhage.

## Packaging and labeling

The Ommaya reservoir and ventricular catheter are each received in separate packaging from their respective companies (most often Natus and Integra, respectively). Packaging is sterile and is only opened immediately prior to implantation.

## Receiving, storage, distribution and return

### Device acquisition

The Ommaya reservoir and ventricular catheter are both obtained directly from the companies (typically Natus and Integra, respectively).

### Storage

Ommaya reservoirs and ventricular catheters are stored at room temperature in their sterile packaging.

### Distribution of study device

The Ommaya and ventricular catheter are retrieved on the day of surgery from the designated research implant storage area by the neurosurgical research team. Packages are inspected for damage. Ommaya reservoirs and ventricular catheters are not utilized if damaged or if the sterile packaging is broken. A device accountability log is utilized for each device to trace the lot number and expiration date of each implanted device. This information is also written on the case report form utilized in each case.

# Study Procedures

Enrolled patients will undergo the consented biopsy as per established routine neurosurgical technique. Details of the biopsy technique are left to the treating physician’s discretion but will not be meaningfully altered due to the reservoir placement or due to participation in the research study. If in the surgeon’s opinion, anatomic, positioning, clinical, logistical or other factors encountered during surgery discourage placement of an Ommaya reservoir, no reservoir will be placed, and the reason will be documented.

**Ommaya reservoir placement during biopsy**

Ommaya reservoir placement will be performed via standard neurosurgical technique, with details at the discretion of the surgeon, except as indicated. The following provides a typical workflow. Required items will be documented on the case report form checklist.

1. The surgical listing will include placement of Ommaya reservoir for research (required).
2. Written consent by the patient to participate in the Ommaya reservoir protocol will be personally confirmed and documented by the surgeon (required).
3. In addition to the clinically indicated surgical plan for the brain biopsy, an appropriate trajectory to the lateral ventricle will be planned for placement of the ventricular catheter, taking care to avoid vascular structures and sulci. Typically, an ipsilateral frontal or parietal trajectory will be deployed, though contralateral placement is acceptable at the discretion of the surgeon for reasons of anatomy, cosmesis, safety, or surgical workflow.
4. The intended entry site will be confirmed using the neuronavigation system and used and used to plan a u-shaped incision that does not cross the planned location of the Ommaya reservoir, or interfere with any possible future craniotomy, if potentially applicable.
5. No changes will be made to the standard use of pre-operative antibiotic.
6. After use of local anesthetic, any unused local will be removed from the field (required).
7. If a patient has a known glioma and is undergoing biopsy for concern for recurrence an Ommaya will be placed without waiting for intra-op pathology results. In this scenario, an Ommaya will be useful for longitudinal monitor even if the recurrence is not active. If the patient has a known CNS malignancy but the specific type is not known, an Ommaya will be placed intra-operatively without waiting for preliminary pathology results. For these cases an Ommaya will be useful for longitudinal monitoring. If a patient has a lesion where there is uncertainty as to whether it is or is not a malignancy of any type, the surgical team will wait for preliminary pathology results before placing an Ommaya. Waiting for preliminary pathology results may extend the time under anesthesia for approximately 20 minutes. A burr hole will be made, a small dural opening made, and the ventricular catheter advanced to the lateral ventricle with the aid of neuronavigation until CSF is encountered. Up to 10cc of CSF may be recovered from the ventricular catheter and sent for research while the catheter is advanced to the desired ventricular depth. The catheter is sutured to the Ommaya reservoir with silk suture. Copious irrigation is performed. The Ommaya reservoir is injected with a solution of rifampin/gentamicin/vancomycin after placement. The galea and skin are closed in the usual fashion.
8. As an additional precaution in some cases based on clinical judgment, patients may receive 1 week of prophylactic Bactrim ® (1 single-strength (80 mg TMP/400 mg SMX) tablet q.d., PO).
9. The Ommaya reservoir is implanted permanently, except in the case of a severe infection warranting removal, refractory to antibiotics.
10. The Ommaya collections will occur at least twice post-surgery. We plan on accessing the Ommaya through out the patient’s disease course at clinical care visits.

A case report form will be utilized during the surgery to document compliance with study procedures and to record Ommaya reservoir and catheter lot numbers.

Reservoir aspiration

Verbal informed consent is obtained, referencing the prior written informed consent. The dome of the reservoir is palpated to confirm positioning, and depressed 2-3x to ensure the reservoir content reflects current ventricular CSF composition. The scalp is then thoroughly prepared with antiseptic scrub and a sterile field prepared. Using strict sterile technique, a 25-gauge butterfly needle or similar, the reservoir is percutaneously accessed and no more than 20cc of CSF removed.

Intra-operative baseline lumbar puncture (optional):

Most patients with brain tumors do not have an Ommaya reservoir or other CSF access device, meaning that the only routine access to CSF biomarkers would be via a lumbar puncture. However, it is unclear to what extent lumbar CSF accurately reflects intracranial CSF. If the patient is willing to consent to an intra-operative lumbar puncture while under anesthesia the paired samples can be utilized to determine the relative utility of intracranial versus lumbar CSF for recovery of tumor-associated biomarkers. Patients who prefer to decline the intra-op baseline LP may do so by checking the “no” box on the consent form. By checking the “yes” box, the patient consents to undergoing an intra-operative LP. Even if the patient checks “yes,” the surgeon may elect to abstain from performing an intra-operative LP for any reason, including but not limited to considerations of surgical efficiency, safety, comfort, patient history, anatomy, and/or expected scientific value. Lumbar puncture if performed, will utilize a spinal needle guided by anatomic landmarks, and follow established standard sterile technique.

No more than 20 mL CSF will be withdrawn from each patient during a single day on this protocol, inclusive of lumbar puncture and ventricular fluid collection under a single anesthetic. The samples will be kept on ice for transport, and then processed by the neuro-oncology biorepository personnel.

**STUDY CALENDAR / TEST SCHEDULE:**

|  | Within 21 days prior to registration | Day 0 during biopsy | Day 0-After biopsy | < 7 days after biopsy | > 7 days after biopsy |
| --- | --- | --- | --- | --- | --- |
| Informed consent | X |  |  |  |  |
| Surgical planning MRI | X |  |  |  |  |
| Demographics, Medical history | X |  |  |  |  |
| Physical and neurological exam, ECOG PS | X |  |  | X^1^ | X^2^ |
| Ommaya Reservoir implantation |  | X^R^ |  |  |  |
| Intra-op lumbar puncture CSF collection (optional) |  | X^R^ |  |  |  |
| Intent for CSF collection |  | X |  |  | X^3^ |
| Adverse event evaluation |  | X | X | X^1^ | X^2^ |
| Head CT | X |  |  | X |  |
| CSF collection |  |  |  |  | X |

Note:

R indicates: Research funded,

1 indicates: Neurological deficits and adverse events should be evaluated within 7 days from the date of surgery. If there are no new deficits, further evaluation is not needed.

2 indicates: Will be performed ONLY IF new (not present before surgery) neurological deficits are detected < 7 days after the date of surgery, neurological deficits should be re-evaluated at > 42 days post operatively to determine persistence.

3 indicates: Possible specific time points for CSF extraction include a) after completion of therapy, prior to tumor recurrence; b) if applicable, at time of clinically suspected tumor recurrence, but are left to the discretion of the study team. No tapping will occur while the patient is still inpatient from their biopsy.

# Statistical Plan

## Sample size determination

This is an exploratory, biobanking-based study. As such, no specific sample size determinations have been performed. Once safety and feasibility have been determined with the first ten patients, more patients may be enrolled with a sample size modification to this trial.

## Statistical methods

**Descriptive statistics**

Baseline values for demographic, clinical, and outcome variables (primary and secondary) will be compiled for all participants.

**Hypothesis:** Placement of Ommaya reservoirs at the time of biopsy will be feasible, enabling longitudinal collection and analysis of CSF samples.

The number of samples collected for each patient will be recorded. Methods for biomarker-based assays, and analyses thereof, will depend on the biomarker. Metabolomics, proteomics, and cell-free DNA, among other types of assays, will be performed; analysis will most commonly be based on fold-change over time between longitudinally obtained samples.

## Subject population(s) for analysis

All patients will be included in analyses as appropriate based on the biomarker analysis performed.

# Safety and Adverse Events

All adverse events occurring during the study, including those not meeting the criteria of an Unanticipated Adverse Device Effect (UADE) will be recorded on the appropriate case report form. Records of these events will be maintained, and reports submitted to the IRB according to the regulatory requirements. Expected clinical adverse events and nonsignificant (not serious) clinical adverse events will not be reported. Expected clinical adverse events and anticipated adverse device effects are those listed in Section 1.5.2.

## Definitions

**Unanticipated Adverse Device Effect (UADE)**

A UADE is any serious adverse effect on health or safety or any life-threatening problem or death caused by, or associated with, a device if that effect, problem or death was not previously identified in nature, severity, or degree of incidence in the investigational plan or IDE application (including a supplementary plan or application), or any other unanticipated serious problem associated with a device that relates to the rights, safety, or welfare of subjects.

**Adverse Effect (Event)**

Any untoward medical occurrence in a subject involved in clinical study of an investigational device; regardless of the causal relationship of the problem with the device or, if applicable, other study related treatment(s).

**Associated with the investigational device:** There is a reasonable possibility that the adverse effect may have been caused by the investigational device.

**Life-threatening adverse effect:** Any adverse effect that places the subject, in the view of either the investigator, at immediate risk of death from the effect as it occurred. It does not include a reaction that, had it occurred in a more severe form, might have caused death.

**Serious adverse effect:** An adverse effect is considered “serious” if, in the view of either the investigator, it results in any of the following outcomes:

- death
- a life-threatening AE
- inpatient hospitalization or prolongation of existing hospitalization
- a persistent or significant disability/incapacity
- a congenital anomaly/birth defect.

**Unanticipated adverse effect:** Any adverse effect, the nature, specificity, severity, or frequency of which is not consistent with the risk information in the clinical study protocol or elsewhere in the current IDE application.

**General Physical Examination Findings**

At screening, any clinically significant abnormality should be recorded as a preexisting condition. At the end of the study, any new clinically significant findings/abnormalities that meet the definition of an adverse event must also be recorded and documented as an adverse event.

**Hospitalization, Prolonged Hospitalization or Surgery**

Any adverse event that results in hospitalization or prolonged hospitalization should be documented and reported as an unanticipated adverse device effect unless specifically instructed otherwise in this protocol. Any condition responsible for surgery should be documented as an adverse event if the condition meets the criteria for an adverse event.

Neither the condition, hospitalization, prolonged hospitalization, nor surgery are reported as an adverse event in the following circumstances:

- Hospitalization or prolonged hospitalization for diagnostic or elective surgical procedures for a preexisting condition. Surgery should **not** be reported as an outcome of an adverse event if the purpose of the surgery was elective or diagnostic and the outcome was uneventful.
- Hospitalization or prolonged hospitalization for therapy of the target disease of the study, unless it is a worsening or increase in frequency of hospital admissions as judged by the clinical investigator.

**Post-study Adverse Event**

All unresolved adverse events should be followed by the investigator until the events are resolved, the subject is lost to follow-up, or the adverse event is otherwise explained. At the last scheduled visit, the local investigator should instruct each subject to report, to the local investigator, any subsequent event(s) that the subject, or the subject’s personal physician, believes might reasonably be related to participation in this study.

**Preexisting condition**

A preexisting condition is one that is present at the start of the study. A preexisting condition should be recorded as an adverse event if the frequency, intensity, or the character of the condition worsens during the study period.

**Unanticipated problems involving risk to subjects or others (UPIRTSO)**

Any unanticipated problem or adverse event that meets all of the following three criteria:

- Serious: Serious problems or events that results in significant harm, (which may be physical, psychological, financial, social, economic, or legal) or increased risk for the subject or others (including individuals who are not research subjects). These include: (1) death; (2) life threatening adverse experience; (3) hospitalization - inpatient, new, or prolonged; (4) disability/incapacity - persistent or significant; (5) birth defect/anomaly; (6) breach of confidentiality and (7) other problems, events, or new information (i.e. publications, DSMB reports, interim findings, product labeling change) that in the opinion of the local investigator may adversely affect the rights, safety, or welfare of the subjects or others, or substantially compromise the research data, **AND**
- Unanticipated: (i.e. unexpected) problems or events are those that are not already described as potential risks in the protocol, consent document, not listed in the Investigator’s Brochure, or not part of an underlying disease. A problem or event is "unanticipated" when it was unforeseeable at the time of its occurrence. A problem or event is "unanticipated" when it occurs at an increased frequency or at an increased severity than expected, **AND**
- Related: A problem or event is "related" if it is possibly related to the research procedures

**Adverse event reporting period**

Ommaya reservoir-related adverse events are likely to happen within 30 days of placement. However, accessing the reservoir may also add a very small but real risk of infection. As such, in addition to standard monitoring as described above, each patient’s chart will be reviewed after at least 60 days from the most recent reservoir access to specifically evaluate for any evidence of CNS infection that could be associated with accessing the reservoir.

## Recording of adverse events

At each contact with the subject, the investigator must seek information on adverse events by specific questioning and, as appropriate, by examination. Study subjects will be routinely questioned about adverse effects at study visits. Information on all adverse events should be recorded immediately in the source document, and in the appropriate adverse event section of the case report form (CRF). All clearly related signs, symptoms, and abnormal diagnostic, laboratory or procedure results should be recorded in the source document.

All adverse events occurring during the study period must be recorded. All observed or volunteered adverse effects (serious or non-serious) and abnormal test findings, regardless of the treatment group if applicable or suspected causal relationship to the investigational device or if applicable other study treatment or diagnostic product(s) will be recorded in the subjects’ case history. For all adverse effects sufficient information will be pursued and or obtained as to permit an adequate determination of the outcome, an assessment of the casual relationship between the adverse effect and the investigational device or, if applicable other study treatment or diagnostic product. The clinical course of each event should be followed until resolution, stabilization, or until it has been ultimately determined that the study treatment or participation is not the probable cause. Serious adverse events that are still ongoing at the end of the study period must be followed up, to determine the final outcome. Any serious adverse event that occurs after the study period and is considered to be at least possibly related to the study treatment or study participation should be recorded and reported immediately.

Adverse event monitoring and reporting is a routine part of every clinical trial. Accordingly, investigators will perform appropriate AE monitoring according to the following established standard protocol: first, identify and grade the severity of the event using a copy of the CTCAE v5.0. Next, determine whether the event is expected or unexpected and if the adverse event is related to the medical treatment or procedure. With this information, determine whether an adverse event should be reported as an expedited report or as part of the routinely reported clinical data. Important: All AEs reported via expedited mechanisms must also be reported via the routine data reporting mechanisms defined by the protocol.

| **Category (CTCAE)** | **Adverse Event** | **Baseline** | **Intra-operative** | **< 7 days postoperative** | **60 days from placement or most recent access** |
| --- | --- | --- | --- | --- | --- |
| Nervous System Disorders | Intracranial hemorrhage | X | X | X |  |
|  | Other, persistent neurological deficit | X |  | X | X  (only if new neurological deficit identified < 7 days postoperatively) |
|  | Infection of cranial wound or Ommaya reservoir; meningitis |  |  | X | X |
|  | Other persistent deficit related to lumbar puncture^1^ |  |  | X |  |

*Although subject will no longer be active on the study schedule after their standard of care 3 month post-op visit, the Ommaya reservoir site will continue to be assessed for infection during routine clinical follow-up. Signs of infection will be documented and treated per clinician’s recommendation.

^1^AEs for lumbar puncture may include persistent local lumbar hemorrhage, intracranial hypotension, persistent radicular pain, infection of CNS space.

Causality and severity assessment

The investigator will promptly review documented adverse effects and abnormal test findings to determine 1) if the abnormal test finding should be classified as an adverse effect; 2) if there is a reasonable possibility that the adverse effect was caused by the investigational device or other study treatments; and 3) if the adverse effect meets the criteria for a serious adverse effect.

Assessment of Attribution

When assessing whether an adverse event is related to the insertion and/or use of CSF access (Ommaya and/or LP), the following attribution categories will be utilized:

Definite - The adverse event is clearly related to insertion and/or use of CSF access.

Probable - The adverse event is likely related to insertion and/or use of CSF access.

Possible - The adverse event may be related to insertion and/or use of CSF access.

Unlikely - The adverse event is doubtfully related to insertion and/or use of CSF access.

Unrelated - The adverse event is clearly NOT related to insertion and/or use of CSF access.

## Reporting of unanticipated adverse device effects and unanticipated problems

When an adverse event has been identified, the study team will take appropriate action necessary to protect the study participant and then complete the Study Adverse Event Worksheet and log. The investigator will evaluate the event and determine the necessary follow-up and reporting required.

The investigator will promptly review documented Unanticipated Adverse Device Effects and as necessary shall report the results of such evaluation to IRB within 5 working days of initial notice of the effect. Thereafter the investigator will submit such additional reports concerning the effect as requested.

### Investigator reporting, notifying IRB

All grade 4 or 5 unexpected adverse events with an attribution of possible, probable or definite will be reported using the Adverse Event Expedited Report Form within two business days.

Non-reportable events will be reported at the time of IRB continuing review. This will include a brief narrative summary describing the nature, type and frequency of events that have occurred since the last progress report.

Expedited and routine reports are to be completed within the timeframes and via the mechanisms specified. All expedited AE reports must also be sent to the local Institutional Review Board (IRB) according to local IRB’s policies and procedures.

The investigator will report to the IRB any UPIRTSOs and Non-UPIRTSOs according to the IRB Policy and Procedures.

**Deviations from the investigational plan.**

The investigator shall notify the IRB of any deviation from the investigational plan to protect the life or physical well-being of a subject in an emergency. Such notice shall be given as soon as possible, but in no event later than 5 working days after the emergency occurred. Except in such an emergency, prior approval by the investigator is required for changes in or deviations from a plan, and if these changes or deviations may affect the scientific soundness of the plan or the rights, safety, or welfare of human subjects, IRB notification in accordance is required.

## Stopping Rules

In the event of a grade 4 or 5 adverse event, no further patients will be enrolled until the cases have been reviewed by the DSMB and a plan has been developed for future patients. All adverse events, independent of grade, will be reviewed by the DSMB at meetings.

## Medical Monitoring

It is the responsibility of the investigator to oversee the safety of the study. This safety monitoring will include careful assessment and appropriate reporting of adverse events as noted above, as well as the construction and implementation of a site data and safety-monitoring plan (see Section 10 Auditing, Monitoring and Inspecting). Medical monitoring will include a regular assessment of the number and type of serious adverse events.

### Internal Data and Safety Monitoring Board

The Neurosurgery data and safety monitoring board (DSMB) will initially review the protocol and they will note in their minutes when to meet next to oversee this study. The study data and safety monitoring will be reviewed by a DSMB consisting of a minimum of three members. The board will meet after the third, sixth, and tenth patients have completed their surgery. The DSMB has a written charter attached with complete details.

# Data Handling and Record Keeping

## Confidentiality

Information about study subjects will be kept confidential and managed according to the requirements of the Health Insurance Portability and Accountability Act of 1996 (HIPAA). Those regulations require a signed subject authorization informing the subject of the following:

- What protected health information (PHI) will be collected from subjects in this study
- Who will have access to that information and why
- Who will use or disclose that information
- The rights of a research subject to revoke their authorization for use of their PHI.

In the event that a subject revokes authorization to collect or use PHI, the investigator, by regulation, retains the ability to use all information collected prior to the revocation of subject authorization. For subjects that have revoked authorization to collect or use PHI, attempts should be made to obtain permission to collect at least vital status (long term survival status that the subject is alive) at the end of their scheduled study period.

## Source documents

Source data comprise all information, original records of clinical findings, observations, or other activities in a clinical trial necessary for the reconstruction and evaluation of the trial. Source data are contained in source documents. Examples of these original documents, and data records include: hospital records, clinical and office charts, laboratory notes, memoranda, subjects’ diaries or evaluation checklists, pharmacy dispensing records, recorded data from automated instruments, copies or transcriptions certified after verification as being accurate and complete, microfiches, photographic negatives, microfilm or magnetic media, x-rays, subject files, and records kept at the pharmacy, at the laboratories, and at medico-technical departments involved in the clinical trial. When applicable, information recorded on the CRF shall match the Source Data recorded on the Source Documents.

## Case Report Forms

A Case Report Form (CRF) will be completed for each subject enrolled into the clinical study. The investigator will review, approve and sign/date each completed CRF; the investigator’s signature serving as attestation of the investigator’s responsibility for ensuring that all clinical and laboratory data entered on the CRF are complete, accurate and authentic.

The study case report form (CRF) is the primary data collection instrument for the study. All data requested on the CRF must be recorded. All missing data must be explained. If a space on the CRF is left blank because the procedure was not done or the question was not asked, write “N/D”. If the item is not applicable to the individual case, write “N/A”. All entries should be printed legibly in black ink. If any entry error has been made, to correct such an error, draw a single straight line through the incorrect entry and enter the correct data above it. All such changes must be initialed and dated. Do not obliterate, erase, or use “white-out” for errors. For clarification of illegible or uncertain entries, print the clarification above the item, then initial and date it. If the reason for the correction is not clear or needs additional explanation, neatly include the details to justify the correction.

**Data Management, Processing, and Security**

Patient information and clinical variables will be maintained by the PI and study team on a secured data sheet. Sample information will also be found in BOSS after the samples have been acquisitioned by BAP. All analyses performed on samples obtained from this study will also be maintained securely on an internal server. All samples receive a de-identified code that is utilized when the samples are sent for analyses, both inside and outside of the institution.

**Data Quality Assurance**

The PI and clinical research coordinator will ensure that all data in the database accurately reflect data on the CRFs. All CRFs will be maintained throughout the duration of the study and scanned electronically following signature by a study team member into the study binder to ensure data integrity.

**Data Clarification Process**

The PI and appropriate members of the study will resolve any data queries associated with the study.

## Records Retention

The investigator will maintain records and essential documents related to the conduct of the study. These will include subject case histories and regulatory documents.

The investigator will retain the specified records and reports during the study per institutional policies.

# Study Monitoring, Auditing, and Inspecting

## Study Monitoring Plan

This investigator study will be monitored on a routine basis during the conduct of the trial. The Office of Research Regulatory Support will provide assistance to the investigator clinical monitoring for the trial as a service for the investigator. Clinical trial monitoring requires review of the study data generated throughout the duration of the study to ensure the validity and integrity of the data along with the protection of human research subjects.

The investigator will allocate adequate time for such monitoring activities. The Investigator will also ensure that the monitor or other compliance or quality assurance reviewer is given access to all the study-related documents and study related facilities (e.g., pharmacy, diagnostic laboratory, etc.), and has adequate space to conduct the monitoring visit.

## Auditing and Inspecting

The investigator will permit study-related monitoring, audits, and inspections by the IRB of all study related documents (e.g., source documents, regulatory documents, data collection instruments, study data etc.). The investigator will ensure the capability for inspections of applicable study-related facilities (e.g., pharmacy, diagnostic laboratory, etc.). Participation as a investigator in this study implies acceptance by applicable compliance offices.

# Ethical Considerations

This study is to be conducted according to United States government regulations and Institutional research policies and procedures.

This protocol and any amendments will be submitted to a properly constituted local Institutional Review Board (IRB), in agreement with local legal prescriptions, for formal approval of the study. The decision of the IRB concerning the conduct of the study will be made in writing to the investigator before commencement of this study.

All subjects for this study will be provided a consent form describing this study and providing sufficient information for subjects to make an informed decision about their participation in this study. This consent form will be submitted with the protocol for review and approval by the IRB for the study. The formal consent of a subject, using the approved IRB consent form, must be obtained before that subject undergoes any study procedure. The consent form must be signed and dated by the subject or the subject’s legally authorized representative, and the individual obtaining the informed consent.

# Study Finances

## Funding Source

## Conflict of Interest

Any study team member who has a conflict of interest with this study (patent ownership, royalties, or financial gain greater than the minimum allowable by their institution, etc.) must have the conflict reviewed by a properly constituted Conflict of Interest Committee with a Committee-sanctioned conflict management plan that has been reviewed and approved by the study investigator prior to participation in this study.

# Publication Plan

The PI holds the primary responsibility for publication of results of the study. The study will be registered to ClinicalTrials.gov and all associated information will be maintained accurately.

# References

1 Stupp, R. *et al.* Radiotherapy plus concomitant and adjuvant temozolomide for glioblastoma. *N Engl J Med* **352**, 987-996, doi:10.1056/NEJMoa043330 (2005).

2 Claus, E. B. *et al.* Survival and low-grade glioma: the emergence of genetic information. *Neurosurg Focus* **38**, E6, doi:10.3171/2014.10.Focus12367 (2015).

3 Arora, R. S. *et al.* Age-incidence patterns of primary CNS tumors in children, adolescents, and adults in England. *Neuro Oncol* **11**, 403-413, doi:10.1215/15228517-2008-097 (2009).

4 Diwanji, T. P., Engelman, A., Snider, J. W. & Mohindra, P. Epidemiology, diagnosis, and optimal management of glioma in adolescents and young adults. *Adolesc Health Med Ther* **8**, 99-113, doi:10.2147/AHMT.S53391 (2017).

5 Ostrom, Q. T. *et al.* CBTRUS statistical report: primary brain and central nervous system tumors diagnosed in the United States in 2007-2011. *Neuro Oncol* **16 Suppl 4**, iv1-63, doi:10.1093/neuonc/nou223 (2014).

6 Boele, F. W. *et al.* Long-term wellbeing and neurocognitive functioning of diffuse low-grade glioma patients and their caregivers: A longitudinal study spanning two decades. *Neuro-Oncology* **25**, 351-364, doi:10.1093/neuonc/noac185 (2022).

7 Mellinghoff, I. K. *et al.* Vorasidenib in IDH1- or IDH2-Mutant Low-Grade Glioma. *New England Journal of Medicine*, doi:10.1056/NEJMoa2304194 (2023).

8 Müller Bark, J., Kulasinghe, A., Chua, B., Day, B. W. & Punyadeera, C. Circulating biomarkers in patients with glioblastoma. *British Journal of Cancer* **122**, 295-305, doi:10.1038/s41416-019-0603-6 (2020).

9 Schmid, D. *et al.* Diagnostic biomarkers from proteomic characterization of cerebrospinal fluid in patients with brain malignancies. *J Neurochem* **158**, 522-538, doi:10.1111/jnc.15350 (2021).

10 Ommaya, A. K. Subcutaneous reservoir and pump for sterile access to ventricular cerebrospinal fluid. *Lancet* **2**, 983-984, doi:10.1016/s0140-6736(63)90681-0 (1963).

11 Zubair A, D. J. O. *Ommaya Reservoir*, <Available from: <https://www.ncbi.nlm.nih.gov/books/NBK559011/>> (2020 Jan).

12 Yang, X. T., Feng, D. F., Zhao, L., Sun, Z. L. & Zhao, G. Application of the Ommaya Reservoir in Managing Ventricular Hemorrhage. *World Neurosurg* **89**, 93-100, doi:10.1016/j.wneu.2015.12.040 (2016).

13 Bergman, J. *et al.* Intrathecal treatment trial of rituximab in progressive MS: An open-label phase 1b study. *Neurology* **91**, e1893-e1901, doi:10.1212/wnl.0000000000006500 (2018).

14 Blaney, S. M. *et al.* Phase I clinical trial of mafosfamide in infants and children aged 3 years or younger with newly diagnosed embryonal tumors: a pediatric brain tumor consortium study (PBTC-001). *J Clin Oncol* **23**, 525-531, doi:10.1200/jco.2005.06.544 (2005).

15 Blaney, S. M. *et al.* Intrathecal mafosfamide: a preclinical pharmacology and phase I trial. *J Clin Oncol* **23**, 1555-1563, doi:10.1200/jco.2005.06.053 (2005).

16 Bernardi, R. J. *et al.* Phase I clinical trial of intrathecal gemcitabine in patients with neoplastic meningitis. *Cancer Chemother Pharmacol* **62**, 355-361, doi:10.1007/s00280-007-0601-x (2008).

17 Wilson, R., Osborne, C. & Halsey, C. The Use of Ommaya Reservoirs to Deliver Central Nervous System-Directed Chemotherapy in Childhood Acute Lymphoblastic Leukaemia. *Paediatr Drugs* **20**, 293-301, doi:10.1007/s40272-018-0298-9 (2018).

18 Szvalb, A. D. *et al.* Ommaya reservoir-related infections: Clinical manifestations and treatment outcomes. *Journal of Infection* **68**, 216-224, doi:<https://doi.org/10.1016/j.jinf.2013.12.002> (2014).

19 Mead, P. A., Safdieh, J. E., Nizza, P., Tuma, S. & Sepkowitz, K. A. Ommaya reservoir infections: a 16-year retrospective analysis. *J Infect* **68**, 225-230, doi:10.1016/j.jinf.2013.11.014 (2014).
