# Supplementary material for: A hitchhiker’s guide to cerebrospinal fluid biomarkers for neuro-oncology
Source: Neuro Oncol. 2024 Dec 30;27(5):1165–79. doi: 10.1093/neuonc/noae276 (PMC12187377; doi:10.1093/neuonc/noae276)
Supplement: noae276_suppl_Supplementary_Materials [file noae276_suppl_supplementary_materials.zip › noae276_suppl_materials/CSFreview_Ommaya-at-resection_protocol_supp_final.docx]

**OMMAYA RESERVOIR PLACEMENT FOR PROSPECTIVE BIOMARKER COLLECTION**

**Objectives**

Primary Objectives:

1. To determine the safety and feasibility of intra-operative Ommaya Reservoir placement during a clinically indicated tumor surgery, to facilitate a longitudinal access to CSF for analysis of exploratory and future tumor biomarkers for individualized monitoring
2. To obtain CSF from patients with brain tumors for biomarker discovery.

Exploratory/Correlative objectives:

1. Banking of CSF for future analyses.

**Background / scientific rationale**

Glioma

Glioblastoma (GBM) remains the most common and fatal primary brain tumor in adults. Preclinical work depends largely upon preclinical models of patient-derived xenografts and genetically engineered models as well as various in vitro and ex vivo culture models. Such models carry limitations of immunosuppression and/or species-dependent differences in the tumor and microenvironment. Meanwhile, primary central nervous system tumors are the third most common cancer and the third most common cause of cancer death in adolescents and young adults.^1,2^ Lower-grade gliomas (LGG) are the most common malignant primary CNS tumor in younger patients.^3-5^ Although patients with LGG have relatively prolonged survival, these tumors strike early in life, cause significant neurological morbidity, and are eventually fatal.^4,6^ Despite the dire need to develop novel therapies for LGG, reliable preclinical models are not available and clinical trials targeting these tumors are infrequent. Meaningful results from such studies can take 10-20 years to materialize. When clinical trial results do emerge for glioma, the amount of mechanistic feedback available is typically limited. Unless post-hoc analysis suggests a promising subgroup, failed drugs may be abandoned, with little understanding of why they failed. For all of these reasons, novel strategies are needed that can efficiently and accurately predict the response to treatment, reveal mechanisms of resistance within human tumors, and inform optimal therapeutic direction within the lifetime of individual patients.

Ommaya/Rickham Reservoir

The Ommaya or Rickham reservoir was first introduced in 1963 as a means to allow access to intraventricular cerebrospinal fluid (CSF).^7^ Current indications include: administration of intrathecal chemotherapy, antibiotherapy or opioid medication, chronic drainage of CSF in infants with intraventricular hemorrhage, chronic aspiration of treatment resistant tumor cyst, and aspiration of residual subdural hematomas^8^. This device has been used in a number of clinical trials and retrospective studies which have demonstrated efficiency and device safety.^9-14^ ORs have also been used in pediatric cases as a means to reduce complications derived from ventriculoperitoneal shunt placement.^15^

Biomarkers for CNS malignancies

Liquid biopsies of the CSF demonstrate that tumor DNA is more accurately represented in CSF than in plasma.^16^ Additionally, plasma levels of D-2-hydroxyglutarate, an oncometabolite of IDH-mutant gliomas, are not reliably detectable in human patients with glioma, despite being an important prognostic marker of other IDH mutant tumors^17^. In contrast, D-2-HG levels in IDH-mutant CSF are highly elevated.^16^ Identification of other important biomarkers such as O^6^-methylguanine-DNA methyltransferase (*MGMT*) promoter methylation promoter methylation, has also found to be more sensitive in CSF than in plasma.^18^ In addition, certain biomarkers such as cell-free tumor DNA (cfDNA)^19^ as well as microRNA, are more readily detected in CSF than plasma. For example mi-R21 in CSF of patients with glioma aided in the differentiation of GBM progression versus pseudoprogression.^20^ In addition, cfDNA levels in CSF strongly correlate with critical clinical prognostic factors including MRI features of aggressiveness, tumor enhancement, tumor volume, as well as tumor dissemination.^19^ CSF analysis also provides a source for other relevant structures such extracellular vesicles (EVs) containing exosomal miRNA and proteins. A number of protein biomarkers have been demonstrated to have diagnosis and therapy response value in patients with brain tumors including gliomas, medulloblastomas or meningiomas.^21^

**Rationale for study**

We hypothesize that longitudinal CSF access can provide pragmatic and mechanistic insights regarding tumor identity, behavior, and efficacy of candidate therapies *in vivo*. The identification of biomarkers in CSF is rapidly emerging as a promising minimally invasive approach for monitoring tumor evolution and response to therapy. To date, limited longitudinal access to CSF has provided a barrier for studies to evaluate the utility of this resource. This protocol will provide practical experience to our neurosurgical team utilizing Ommaya reservoirs in the clinical environment, providing access to samples for hypothesis testing and hypothesis generation. Additionally, this protocol will provide our patients with an option to participate in an intra-operative lumbar puncture under anesthesia to obtain baseline, pre-resection CSF, minimizing discomfort while providing a critical data point to identify glioma-associated biomarkers prior to resection at maximal tumor burden.

**Patient eligibility**

Inclusion Criteria

- Age > 18 years.
- Clinical and radiographic evidence suggesting a diagnosis of a brain tumor.
- Planned neurosurgical procedure resection of suspected or previously diagnosed brain tumor as part of routine clinical care.
- Willing to undergo neurosurgical resection at the institution.
- Willingness of the patient or an authorized representative to provide informed consent.
- Patient is willing to have their Ommaya sampled on at least 2 future occasions.
- Patients is willing to have CSF banked through the neuro-oncology biorepository (requires a separate signature).

Exclusion Criteria:

- Vulnerable populations including pregnant women, prisoners and individuals <18 years old.
- Patients who are not appropriate candidates for surgery due to current or past medical history or uncontrolled concurrent illness.
- Prior history of any wound infection.
- Any patient who the surgeon feels is not an optimal candidate for Ommaya reservoir placement. Such reasons may (but need not necessarily) include surgical anatomy clinical evidence of significant immunosuppression, and/or elevated risk of wound infection due to diabetes, smoking history, morbid obesity, or any other concerns.
- Any patient for whom a clinical contraindication exists to the intended route of CSF access will be excluded from the Lumbar Puncture portion of this protocol. For example, a patient with a large posterior fossa mass lesion at risk of herniation, or a patient with coagulopathy, or other contraindication to lumbar puncture would not be eligible to participate via use of lumbar puncture for CSF access. These patients would still be eligible for an Ommaya reservoir if they meet the inclusion criteria of the study.

**Study calendar/test schedule**

|  | Within 21 days prior to registration | Day 0 during surgery | Day 0-Afte Surgery | < 7 days after date of surgery | > 42 days after date of surgery |
| --- | --- | --- | --- | --- | --- |
| Informed consent | X |  |  |  |  |
| Surgical planning MRI | X |  |  |  |  |
| Demographics, Medical history | X |  |  |  |  |
| Physical and neurological exam, ECOG PS | X |  |  | X^1^ | X^2^ |
| Ommaya Reservoir implantation |  | X^R^ |  |  |  |
| Intra-op lumbar puncture CSF collection (optional) |  | X^R^ |  |  |  |
| Intent for CSF collection |  | X |  |  | X^3^ |
| Adverse event evaluation |  | X | X | X^1^ | X^2^ |
| MRI brain |  |  |  | X |  |

Note:

R indicates: Research funded,

1 indicates: Neurological deficits and adverse events should be evaluated within 7 days from the date of surgery. If there are no new deficits, the > 42-day assessment is not necessary.

2 indicates: Will be performed only if new (not present before surgery) neurological deficits are detected < 7 days after the date of surgery, neurological deficits should be re-evaluated at > 42 days post operatively to determine persistence.

3 indicates: Possible specific time points for CSF extraction include a) after completion of therapy, prior to tumor recurrence; b) if applicable, at time of clinically suspected tumor recurrence.

Variations and considerations regarding CSF sampling:

- During discussion with the patient in clinic, the provider may elect to obtain additional samples at other times points of potential clinical relevance for disease monitoring. Examples could include, but are not limited to follow-up visits, MRIs appointments, or disease milestones (change in therapy, treatment response, etc.).
- Verbal consent for CSF sampling will be documented as part of the procedure. This note will be generated whenever the Ommaya reservoir is tapped.
- All CSF draws, regardless of time points, will be documented as part of the longitudinal patient monitoring for the trial, and will be evaluated at DSMB meetings, if required for the purpose of this study.
- Failure to obtain a sample at one or more of the intended time points will not exclude the patient from the trial and it will not be considered a deviation.

**Event monitoring / survival follow up**

Event monitoring and survival follow up will not be performed during this clinical trial.

**Patient classification**

Patients will be stratified by the following categories:

- Metastatic tumor vs low grade vs high grade vs other
- Recurrent vs non-recurrent
- Previously untreated, vs previously treated tumors s/p prior radiation and/or alkylating therapy.
- Low grade (WHO Grade 2) vs high grade (WHO Grade 3-4) tumors.
- Placement within tumor cavity vs lateral ventricle.
- Tumor resection cavity contacts the lateral ventricle

**Study procedures**

**Surgical procedures:**

- Enrolled patients will undergo the consented neurosurgical resection as per established routine neurosurgical technique. Details of surgical resection technique are left to the treating physician’s discretion, but will not be meaningfully altered due to the reservoir placement procedure or due to participation in the research study.
- If in the surgeon’s opinion, anatomic, positioning, clinical, logistical or other factors encountered during surgery discourage placement of an Ommaya reservoir, no reservoir will be placed, and the reason documented. Such patients will remain enrolled in the study.

**Operation: Ommaya Reservoir Placement**

Ommaya Reservoir placement will be performed during a planned surgery for tumor. Following the routine portion of the planned procedure, the Ommaya catheter will be placed, typically either within the ventricle or the resection cavity intraventricular using preexisting tumor resection cavity, as appropriate based on surgical anatomy.  Ommaya reservoir will be placed in a location deemed optimal by the surgeon, considering cosmetics and accessibility.  The length of catheter and choice of Ommaya reservoir will be tailored to the surgical anatomy. If it is felt that the resection cavity is likely to collapse over time, a separate incision may be made to place the Ommaya in the ventricular system, requiring passage of the catheter through tissue. It is expected that such placement will add approximately 5-15 minutes to the otherwise planned procedure. In addition, antibiotics will be consistently applied in the surgical field intra-operatively per below, in addition to the following safety measures:

1. Per routine anesthesia procedure, prophylactic cefazolin will be utilized for each case in a weight-based manner with first dose prior to incision and regular intra-operative redosing until closure).
2. After dural closure and prior to opening the Ommaya reservoir and catheter, all surgical team members scrubbed will change their outer gloves. Additionally, a new set of sterile instruments will be utilized for closure and Ommaya placement.
3. The Ommaya reservoir will be injected with and immersed in a solution of rifampin/gentamicin/vancomycin prior to placement.
4. Fill the Ommaya reservoir with gentamycin/vancomycin solution. If the patient has a known or suspected allergy to vancomycin or gentamycin, those antibiotics will not be used, and an alternate antibiotic solution will be utilized based on discussions with pharmacy.
5. Perform betadine and peroxide irrigation of the surgical exposure
6. Utilize 1g Vancomycin powder in the surgical wound prior to closure.
7. As an additional precaution in some cases based on clinical judgment (such as in longer cases), patients may receive 1 week of prophylactic Bactrim ® (1 single-strength (80 mg TMP/400 mg SMX) tablet q.d., PO).

A case report form will be utilized during the surgery to document compliance with study procedures and to record Ommaya reservoir and catheter lot numbers.

Reservoir Aspiration

The dome of the reservoir is palpated to confirm positioning, and depressed 2-3x to ensure the reservoir contents are reflective of the CSF composition. The scalp is then prepared with antiseptic scrub. Using a 25 gauge or thinner needle, the reservoir is pierced and no more than 20cc of CSF removed.

Ommaya reservoirs will not be accessed until at least four weeks following placement.

**Operation: Intra-operative baseline lumbar puncture**

It is critical to obtain a pre-resection CSF sample at baseline, in order to identify the tumor-associated CSF biomarkers that would convey tumor burden and response to therapy. Since CSF is not always feasibly obtainable from the surgical field prior to removal of the tumor, an intra-operative baseline LP ensures this critical data is not lost. Patients who prefer to decline the intra-op baseline LP may do so by checking the “no” box on the consent form. By checking the “yes” box, we would be permitted to perform the LP, though may elect not to do if we feel it is not necessary, practical, or appropriate for any reason on the day of surgery.

The CSF samples will be obtained via lumbar puncture, percutaneous access to a CSF reservoir or collection, or access to an external CSF access device. Up to 20 mL CSF will be withdrawn from each patient at a single timepoint. The samples will be kept on ice for transport, and then processed by the neuro-oncology biorepository personnel.

**Duration of follow up**

Patients will be evaluated by the clinical team on a regular basis as per standard practice during their post-operative hospitalization. Clinical notes will serve as the source of primary source of documentation for any new neurological deficits or adverse events. In the event of new neurological deficits, neurological deficits will be re-evaluated at least 42 days post operatively to determine persistence.

**Adverse Event (AE) reporting and monitoring**

Adverse event monitoring and reporting is a routine part of every clinical trial. Accordingly, investigators will perform appropriate AE monitoring according to the following established standard protocol. First, identify and grade the severity of the event using a copy of the CTCAE v5.0. Next, determine whether the event is expected or unexpected and if the adverse event is related to the medical treatment or procedure. With this information, determine whether an adverse event should be reported as an expedited report or as part of the routinely reported clinical data. All AEs reported via expedited mechanisms must also be reported via the routine data reporting mechanisms defined by the protocol.

Required Routine Reporting

Participating in the trial could slightly prolong the surgery due to insertion of an Ommaya, although from experience this takes less than 15 minutes in most cases. Reservoir creates a theoretical risk of hemorrhage. However, for catheters left in the resection cavity, normal tissue would not be passed, mitigating this risk. Prior studies have demonstrated that infection of an Ommaya reservoir can occur, and typically does so within 30 days of placement or most recent access (usually for administration of intrathecal chemotherapy). As such, we will require monitoring for at least 90 days from the time of surgery to evaluate for placement-associated infections. Infections associated with access to the Ommaya reservoir will continue to be documented as such information is made available through routine clinical follow-up for life. However, since Ommaya reservoir access timing may vary based upon other studies, no specific time points will be mandated beyond 90 days. Pretreatment symptoms / conditions are to be graded at baseline and adverse events to be graded at stated time-points.

| **Category (CTCAE)** | **Adverse Event** | **Baseline** | **Intra-operative** | **< 7 days postoperative** | **> 90 days postoperative*** |
| --- | --- | --- | --- | --- | --- |
| Nervous System Disorders | Intracranial hemorrhage | X | X | X |  |
|  | Other, persistent neurological deficit | X |  | X | X  (only if new neurological deficit identified < 7 days postoperatively) |
|  | Infection of cranial wound or Ommaya reservoir; meningitis |  |  | X | X |
|  | Other persistent deficit related to lumbar puncture^1^ |  |  | X |  |

*Although subject will no longer be active on the study schedule after their standard of care 3-month post-op visit, the Ommaya reservoir site will continue to be assessed for infection during routine clinical follow-up. Signs of infection will be documented and treated per clinician’s recommendation.

^1^AEs for lumbar puncture may include persistent local lumbar hemorrhage, intracranial hypotension, persistent radicular pain, infection of CNS space.

**Adverse event characteristics**

The descriptions and grading scales found in the revised NCI Common Terminology Criteria for Adverse Events (CTCAE) version 5.0 will be utilized for AE reporting. All appropriate treatment areas should have access to a copy of the CTCAE version 5.0. A copy of the CTCAE version 5.0 can be downloaded from the CTEP web site (http://ctep.cancer.gov).

**Assessment of attribution**

When assessing whether an adverse event is related to the insertion and/or use of CSF access (Ommaya and/or LP), the following attribution categories will be utilized:

Definite - The adverse event is clearly related to insertion and/or use of CSF access.

Probable - The adverse event is likely related to insertion and/or use of CSF access.

Possible - The adverse event may be related to insertion and/or use of CSF access.

Unlikely - The adverse event is doubtfully related to insertion and/or use of CSF access.

Unrelated - The adverse event is clearly NOT related to insertion and/or use of CSF access.

**Expected and unexpected adverse event reporting requirements**

All grade 4 or 5 unexpected adverse events with an attribution of possible, probable or definite will be reported using the institutional Adverse Event Expedited Report Form within two business days.

Non-reportable events will be reported at the time of IRB continuing review. This will include a brief narrative summary describing the nature, type, and frequency of events that have occurred since the last progress report.

Expedited and routine reports are to be completed within the timeframes and via the mechanisms specified. All expedited AE reports must also be sent to the local Institutional Review Board (IRB) according to local IRB’s policies and procedures.

**Protocol modifications based on adverse events**

This study does not have any ongoing study interventions. Therefore, there are no pre-specified interruptions or treatment alterations due to adverse events.

**Ancillary treatment/supportive care**

Patients will receive standard full supportive care while on this study.

There are no anticipated adverse effects from study participation that will require supportive care specifically related to the research sampling.

**Descriptive factors**

- Demographic data
- Imaging characteristics
- Anatomical details
- Treatment details

**Statistical considerations**

Overview:

This is a pilot trial designed to assess the feasibility of Ommaya reservoir placement in patients with central nervous system malignancies. Risk factors for complications could correlate with prior surgery, prior and/or future radiation, and chemotherapy. These considerations must be balanced with anticipated increased clinical urgency of biomarker-based feedback from the tumor in patients with higher grade and recurrent lesions. As such, a pilot evaluation of relative risk of Ommaya reservoir placement will be evaluated in each of the following patient populations:

Sample size:

A total of 50 adult patients will be enrolled:

- 10 patients with newly diagnosed suspected or biopsy-proven high-grade glioma
- 10 patients with newly diagnosed suspected or biopsy-proven low-grade glioma
- 10 patients with recurrent high-grade glioma
- 10 patients with recurrent low-grade glioma
- 10 patients with suspected pathology other than glioma.

Accrual rate:

Based on institutional experience, the estimated monthly accrual rate is 3 patients per month, so the estimated period of accrual will be approximately 17 months. Since patients may need to be observed for at least 42 days for recovery of neurological deficits, we anticipate the total study duration to be approximately 24 months for accrual, and 2.5 years for the entire study.

Analysis of primary endpoint:

**SAFETY:** The primary safety endpoint will be assessed by evaluating the proportion of patients who: (2) develop persistent adverse events deemed related (possibly, probably, definitely) to the insertion or use of Ommaya Reservoirs. Adverse events will be measured by CTCAE 5.0.

**FEASIBILITY:** The primary feasibility endpoint will be assessed by evaluating the proportion of enrolled patients for whom Ommaya reservoir is successfully placed at the time of surgery without complication.

**Monitoring**

The study will be reviewed by the PI or co-PI and study statistician on a regular (monthly) basis to monitor for severe adverse events and feasibility problems.

**Data and Safety Monitoring Plan/Board**

The neurosurgery data safety monitor board (DSMB) will initially review the protocol, and they will note in their minutes when to meet next to oversee this study. The study data and safety monitoring will be reviewed by a DSMB consisting of the PI and a minimum of 3 members. The board will meet after the 5th patient has complete the surgery, and again at a time to be determined after the first meeting. The DSMB has a written charter attached with complete details.

**Risks and adverse event stopping rules**

When access to the anterior horn of the ventricle for placement of the OR catheter is not feasible due to tumor location, would imply than a second opening in the skull might be required for placement. In such cases, risk of implantation derived complications would be comparable to those to external ventricular drainage.

Another possible complication lies on infection derived from CSF extraction. A retrospective series of 501 patients specifically evaluation OR specific related infection reporting an OR related infection rate of 8%. However, based on previous experience, in the rare case of infection, symptoms are expected to be mild to moderate with normal laboratory findings; while more symptomatic, serious symptoms from infections such obnubilations, seizures or systemic affection, under the context of Ommaya Reservoir tapping, are rare.^22^

Neurologic deficits should be evaluated within 7 days from the date of surgery. If new (not present before surgery) neurologic deficits are detected within 7 days from the date of surgery, neurologic deficits should be evaluated again at >42 days post-operatively to determine if they are persistent; if there are no new deficits found during the initial evaluation, the >42 days assessment is not necessary.

We do not expect the risk of neurological deficit would be significantly increased by leaving an Ommaya reservoir at the end of surgery. Nevertheless, neurological deficit occurring after surgery will be monitored and individually assessed for the possibility of attribution.

Clinical judgement will be deployed to select patients for the trial who are not at unreasonably high risk of wound infection. However, infection is an established risk of Ommaya reservoir placement and will be monitored accordingly.

In the event of infection, management will be performed in the best clinical judgement of the patients medical team. This may require surgery for removal of the Ommaya reservoir, and/or local or systemic antibiotic therapy.

Risks of lumbar puncture:

There is a small risk of local site hemorrhage related to the shunt tap needle (usually 25 gauge) or LP needle, which is controlled with brief local pressure. During the LP, there is risk of nerve root irritation or injury—usually manifested by a short, acute radicular pain that resolves when the stimulus is removed. Lumbar puncture will be performed when the patient is under anesthesia, minimizing the potential discomfort associated with this procedure. There is a small, but important risk of infection from accessing the CSF space. All procedures are performed with meticulous local skin preparation using and standardized sterile technique to minimize risk. The risk of infection following LP is expected to be well under 1% (Baer ET. *Post-dural puncture bacterial meningitis*. Anesthesiology 2006; 105(2):381; Evans RW. *Complications of Lumbar Puncture*. Neurologic Clinics. 1998(16)1:83-105). Finally, LP may result in temporary post-procedural postural headache that often resolves with hydration, caffeine, and rest within a few days. We expect this risk to be minimal in our patient population as they will be placed under anesthesia as part of their standard of care procedure. In rare remote cases, persistence may require blood patching with an interventional radiologist. These risks are cumulatively quite low and are reasonable to facilitate progress toward identification of biomarkers and insights that may improve standards of care for patients with brain tumors.

**Potential benefits**

There are no expected benefits to the individual participants in the study, as study is only performed to evaluate feasibility of Ommaya reservoir placement for consideration of use in parallel/future tumor biomarker-related studies. Given the long history of Ommaya reservoir use in clinical neurosurgery, and the need for CSF access for individualized biomarker discovery, the associated risk is considered reasonable. If the Ommaya reservoir provides opportunity for the patient’s clinician to access biomarkers that could guide management decisions in the future, the potential for future benefit cannot be excluded. This pilot study represents an important starting point in a series of iterative steps toward an envisioned paradigm of biomarker-guided individualized therapies to evaluate status of disease and efficacy of future therapies. Participating in this process is expected to be of benefit to future patients, and conceivably to the patient themselves, should discoveries advance with adequate speed.

**Early withdrawal of subjects**

Subjects may withdraw at any time for any reason by verbal notification to any member of the surgical or study team. The surgeon may elect to the patient from Ommaya reservoir placement during surgery for any reason, including if optimal surgical access for placement of the Ommaya reservoir is not available, or if the surgeon feels completion of the Ommaya reservoir would unduly delay, prolong, or otherwise interfere with the surgery. Subjects who withdraw or are withdrawn after placement or attempted placement of the Ommaya reservoir will continue to be followed for collection of any data related to potential adverse events.

**Data collection and follow-up for withdrawn subjects**

Subjects who withdraw or are withdrawn after placement or attempted placement of an Ommaya reservoir will continue to be followed for collection of any data related to potential adverse events.

**Inclusion of women and minorities**

This study will be available to all eligible patients regardless of race, gender, or ethnic group. There is no information currently available regarding differential impact of Ommaya reservoir use in subsets defined by gender, race or ethnicity, and there is no reason to expect such differences to exist. Therefore, although the planned analyses will, as always, look for differences based on gender and racial groupings, the sample size is not increased to provide additional power for such subset analyses. Based on prior studies at our institution involving glioma, we expect about 7% of patients will be classified as minorities by race and about 40% of patients to be women.

**References:**

**1.** Brown M, Schrot R, Bauer K, Dodge J. Incidence of first primary central nervous system tumors in California, 2001-2005: children, adolescents and teens. *J Neurooncol.* 2009; 94(2):263-273.

**2.** *Childhood cancer in britain: incidence, survival, and mortality.* Vol 571982.

**3.** Arora RS, Alston RD, Eden TO, Estlin EJ, Moran A, Birch JM. Age-incidence patterns of primary CNS tumors in children, adolescents, and adults in England. *Neuro Oncol.* 2009; 11(4):403-413.

**4.** Diwanji TP, Engelman A, Snider JW, Mohindra P. Epidemiology, diagnosis, and optimal management of glioma in adolescents and young adults. *Adolesc Health Med Ther.* 2017; 8:99-113.

**5.** Ostrom QT, Gittleman H, Liao P, et al. CBTRUS statistical report: primary brain and central nervous system tumors diagnosed in the United States in 2007-2011. *Neuro Oncol.* 2014; 16 Suppl 4:iv1-63.

**6.** Stupp R, Mason WP, van den Bent MJ, et al. Radiotherapy plus concomitant and adjuvant temozolomide for glioblastoma. *The New England journal of medicine.* 2005; 352(10):987-996.

**7.** Ommaya AK. Subcutaneous reservoir and pump for sterile access to ventricular cerebrospinal fluid. *Lancet.* 1963; 2(7315):983-984.

**8.** Zubair A DJO. Ommaya Reservoir. 2020 Jan; Available from: <https://www.ncbi.nlm.nih.gov/books/NBK559011/>. Accessed 7/23/2020, 2020.

**9.** Yang XT, Feng DF, Zhao L, Sun ZL, Zhao G. Application of the Ommaya Reservoir in Managing Ventricular Hemorrhage. *World Neurosurg.* 2016; 89:93-100.

**10.** Bergman J, Burman J, Gilthorpe JD, et al. Intrathecal treatment trial of rituximab in progressive MS: An open-label phase 1b study. *Neurology.* 2018; 91(20):e1893-e1901.

**11.** Blaney SM, Boyett J, Friedman H, et al. Phase I clinical trial of mafosfamide in infants and children aged 3 years or younger with newly diagnosed embryonal tumors: a pediatric brain tumor consortium study (PBTC-001). *J Clin Oncol.* 2005; 23(3):525-531.

**12.** Blaney SM, Balis FM, Berg S, et al. Intrathecal mafosfamide: a preclinical pharmacology and phase I trial. *J Clin Oncol.* 2005; 23(7):1555-1563.

**13.** Bernardi RJ, Bomgaars L, Fox E, et al. Phase I clinical trial of intrathecal gemcitabine in patients with neoplastic meningitis. *Cancer Chemother Pharmacol.* 2008; 62(2):355-361.

**14.** Wilson R, Osborne C, Halsey C. The Use of Ommaya Reservoirs to Deliver Central Nervous System-Directed Chemotherapy in Childhood Acute Lymphoblastic Leukaemia. *Paediatr Drugs.* 2018; 20(4):293-301.

**15.** Bin Nafisah S, Ahmad M. Ommaya reservoir infection rate: a 6-year retrospective cohort study of Ommaya reservoir in pediatrics. *Childs Nerv Syst.* 2015; 31(1):29-36.

**16.** McEwen AE, Leary SES, Lockwood CM. Beyond the Blood: CSF-Derived cfDNA for Diagnosis and Characterization of CNS Tumors. *Front Cell Dev Biol.* 2020; 8:45.

**17.** Zhang L, Sorensen MD, Kristensen BW, Reifenberger G, McIntyre TM, Lin F. D-2-Hydroxyglutarate Is an Intercellular Mediator in IDH-Mutant Gliomas Inhibiting Complement and T Cells. *Clinical Cancer Research.* 2018; 24(21):5381-5391.

**18.** Wang Z, Jiang W, Wang Y, et al. MGMT promoter methylation in serum and cerebrospinal fluid as a tumor-specific biomarker of glioma. *Biomed Rep.* 2015; 3(4):543-548.

**19.** Miller AM, Shah RH, Pentsova EI, et al. Tracking tumour evolution in glioma through liquid biopsies of cerebrospinal fluid. *Nature.* 2019; 565(7741):654-658.

**20.** Shi R, Wang PY, Li XY, et al. Exosomal levels of miRNA-21 from cerebrospinal fluids associated with poor prognosis and tumor recurrence of glioma patients. *Oncotarget.* 2015; 6(29):26971-26981.

**21.** Xiao F, Lv S, Zong Z, et al. Cerebrospinal fluid biomarkers for brain tumor detection: clinical roles and current progress. *American journal of translational research.* 2020; 12(4):1379-1396.

**22.** Szvalb AD, Raad II, Weinberg JS, Suki D, Mayer R, Viola GM. Ommaya reservoir-related infections: Clinical manifestations and treatment outcomes. *Journal of Infection.* 2014; 68(3):216-224.
